# Accessing self-diffusion on nanosecond time and nanometre length scales with minute kinetic resolution

**DOI:** 10.1107/S1600576724003820

**Published:** 2024-06-07

**Authors:** Christian Beck, Felix Roosen-Runge, Marco Grimaldo, Dominik Zeller, Judith Peters, Frank Schreiber, Tilo Seydel

**Affiliations:** a Institut für Angewandte Physik Universität Tübingen Auf der Morgenstelle 10 72076Tübingen Germany; b Institut Max von Laue–Paul Langevin, 71 Avenue des Martyrs, 38042Grenoble, France; c Division of Physical Chemistry, Lund University, Naturvetarvägen 14, 22 100Lund, Sweden; d Department of Biomedical Science, Biofilms-Research Center for Biointerfaces, Malmö University, 205 06Malmö, Sweden; e Université Grenoble Alpes, CNRS, LiPhy, Grenoble, France; f Institut Universitaire de France France; Uppsala University, Sweden; The European Extreme Light Infrastucture, Czechia

**Keywords:** quasielastic neutron spectroscopy, data analysis, self-diffusion, single-crystal monochromators

## Abstract

Neutron spectroscopy uniquely and non-destructively accesses diffusive dynamics in soft and biological matter, including for instance proteins in hydrated powders or in solution, and more generally dynamic properties of condensed matter on the molecular level. Given the limited neutron flux resulting in long counting times, it is important to optimize data acquisition for the specific question, in particular for time-resolved (kinetic) studies. The required acquisition time was recently significantly reduced by measurements of discrete energy transfers rather than quasi-continuous neutron scattering spectra on neutron backscattering spectrometers. Besides this reduction in acquisition times, smaller amounts of samples can be measured with better statistics, and most importantly, kinetically changing samples, such as aggregating or crystallizing samples, can be followed. However, given the small number of discrete energy transfers probed in this mode, established analysis frameworks for full spectra can break down. Presented here are new approaches to analyze measurements of diffusive dynamics recorded within fixed windows in energy transfer, and these are compared with the analysis of full spectra. The new approaches are tested by both modeled scattering functions and a comparative analysis of fixed energy window data and full spectra on well understood reference samples. This new approach can be employed successfully for kinetic studies of the dynamics focusing on the short-time apparent center-of-mass diffusion.

## Abstract 2

A new analysis framework to treat quasi-elastic neutron spectroscopy data recorded at discrete energy transfers has been developed. This new approach can be employed successfully for kinetic studies of diffusive dynamics.

## 1. Introduction

To understand kinetic processes in soft and biological matter evolving on timescales from minutes to hours, such as protein aggregation and protein crystallization, suitable experimental methods have to be developed (Morris *et al.*, 2009 ▸; Meisl *et al.*, 2016 ▸; Sun *et al.*, 2018 ▸; Toprakcioglu *et al.*, 2019 ▸; Bramham & Golovanov, 2022 ▸; Housmans *et al.*, 2023 ▸). In order to obtain a comprehensive picture, following structural evolution is not sufficient and these methods have to include the ability to detect diffusive dynamics on nanosecond time and nanometre length scales commensurate with protein dynamics inside living cells and with protein folding dynamics (Sun *et al.*, 2023 ▸). The successful development of these methods is crucial to address challenges such as combatting protein aggregation diseases (Jarrett & Lansbury, 1993 ▸; Cohen *et al.*, 2012 ▸; Arosio *et al.*, 2014 ▸). Neutron spectroscopy can access essential experimental observables for this endeavor, such as self-diffusion on the molecular level, without the need for specific labels (Grimaldo *et al.*, 2019*a* ▸,*b* ▸), and it is one of the few techniques along with X-ray photon correlation spectroscopy (Girelli *et al.*, 2021*b* ▸) which provide length- and timescale information. However, neutron spectroscopy is a fundamentally signal-limited technique due to physical constraints imposed on the neutron source brightness.

Neutron spectroscopy typically measures the dynamic structure factor *S*(*q*, ω) depending on the scattering vector magnitude *q* [*i.e.* momentum transfer ℏ*q* related to the scattering angle; *q* = (4π/λ)sinθ, where θ is half the scattering angle and λ is the wavelength of the incident radiation] and the energy transfer ℏω. *S*(*q*, ω) represents the Fourier transform of the van Hove correlation function *G*(*r*, *t*) depending on space *r* and time *t* (van Hove, 1954 ▸). The observable time­scales are given by the spectrometer resolution function and maximum energy transfer.

**Figure 1:**
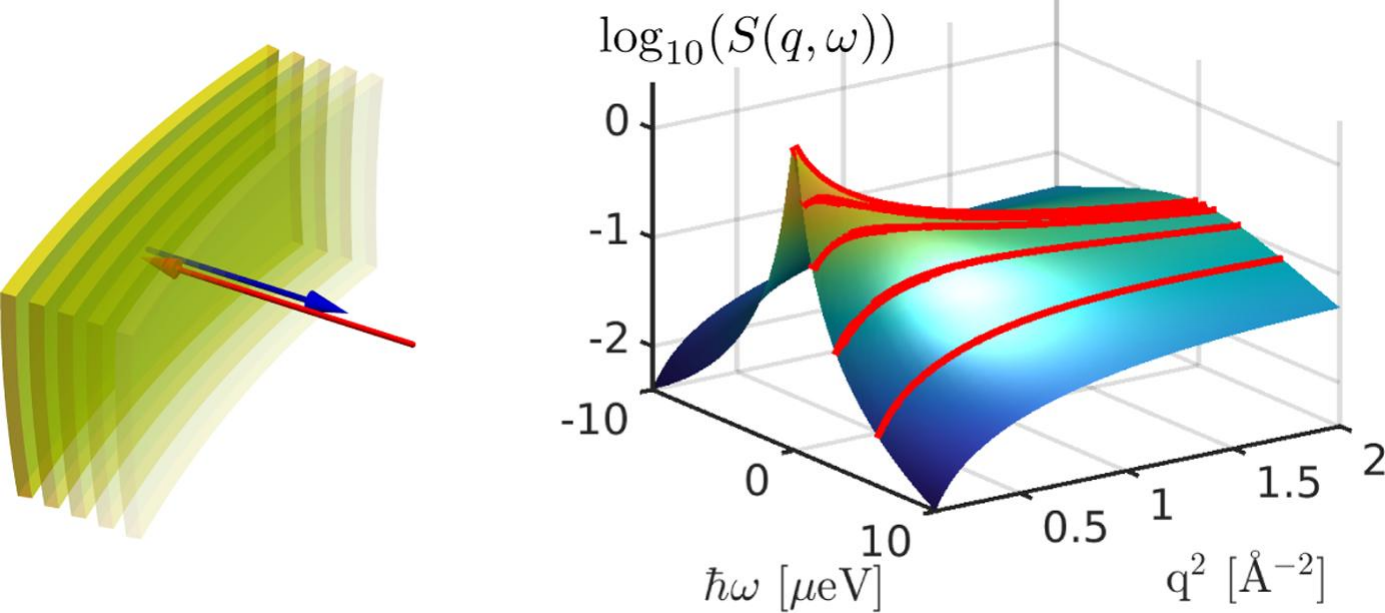
(Left) A schematic representation of a moving monochromator crystal achieving an energy transfer in exact backscattering by a Bragg reflection in a moving reference frame, denoted a Doppler monochromator. [Adapted with permission from Hennig (2011 ▸)]. (Right) A schematic diagram of a QENS spectrum. Depending on the velocity profile (*cf*. Fig. 2) of the Doppler monochromator, full QENS spectra (surface plot) or discrete energy transfers (red lines) can be acquired.

Cold neutron backscattering spectroscopy (NBS) achieves a very high energy resolution of typically better than 1 µeV full width at half-maximum (FWHM) for ℏ*q* up to *q* ≃ 2 Å^−1^ by employing Bragg reflections in exact backscattering from both monochromator and analyzer single crystals. The requirement of backscattering prohibits any change in the crystal Bragg angle for spectral recordings. Therefore, mechanical Doppler drives carrying the monochromator crystal constitute the most common current approach to changing the incident neutron energy by a Bragg reflection in a moving reference frame (Fig. 1 ▸) (Frick & Gonzalez, 2001 ▸; Meyer *et al.*, 2003 ▸; Frick *et al.*, 2010 ▸; Souza *et al.*, 2016 ▸) and are employed in current NBS instruments such as IN16B (Frick *et al.*, 2010 ▸) at the ILL in France, EMU at ANSTO in Australia (Souza *et al.*, 2016 ▸), HFBS at NIST in the USA (Meyer *et al.*, 2003 ▸) and SPHERES at MLZ in Germany (Zamponi & Khaneft, 2015 ▸). Their maximum speed is *v*_max_ ≃ 4.5 m s^−1^, limited by the corresponding acceleration and the monochromator mass (∼1 kg). For comparison, the speed of cold neutrons is ∼630 m s^−1^ at 2 meV energy, and thus the Doppler effect allows the neutron energy to be shifted by a measurable amount.

The mechanical approach is necessitated by the large neutron beam size (∼20 × 30 cm) at the monochromator position due to the focusing optics built into these instruments, rendering the alternative approach of changing the monochromator lattice spacing via temperature (Cook *et al.*, 1992 ▸; Ciampolini *et al.*, 2005 ▸) challenging due to the required temperature homogeneity. Among numerous applications from quantum mechanics to polymer and glass physics, NBS is particularly suited to accessing nanosecond diffusive dynamics (Grimaldo *et al.*, 2019 ▸; Telling, 2020 ▸; Kruteva, 2021 ▸; Peters *et al.*, 2023 ▸; Zheng *et al.*, 2023 ▸; Zhao *et al.*, 2022 ▸). This strength can, for instance, be exploited to measure the diffusion of nanometre-sized soft colloidal particles such as proteins in liquid solutions, which we use as a test case in this article. However, depending on the experimental parameters and sample composition, recording full spectra takes considerable time, typically several hours, to obtain statistically meaningful data.

In this paper, we discuss strategies for more time efficiency, opening up new areas of application of NBS-based quasi-elastic neutron scattering (QENS). We propose analysis frameworks for NBS data acquired at only a few selected discrete energy transfers. In addition to the obvious reduction in acquisition times and enhancement of the signal, kinetically changing samples can be followed, enabling new science.

This article is organized as follows. We first explain the acquisition modes of NBS instruments with a Doppler monochromator. We then focus on the different contributions to the scattering signal at given energy transfers and model the incoherent scattering of protein solutions on the basis of the description of full QENS spectra (Grimaldo *et al.*, 2015*a* ▸). Using modeled data sets, we discuss the non-monotonic contributions to the scattering signal in Section 2. We next focus on established analysis methods (Section 3) and on the application of models developed for full QENS spectra for the analysis of data with distinct energy transfers (Section 4). Subsequently, in Section 5 we present a new approach, resulting in quantitative agreement with the analysis of full QENS spectra. Sections 6 and 7 address the applicability to powder samples and the influence of instrument resolution.

We discuss samples where the scattering signal of interest can be considered spatially incoherent due to the prevalence of the ^1^H isotope with its large incoherent scattering cross section. The D_2_O solvent scattering signal can be subtracted. Different frameworks exist for the analysis of such scattering signals (Doster & Settles, 2005 ▸; Doster & Longeville, 2007 ▸; Doster, 2008 ▸; Grimaldo *et al.*, 2019 ▸; Zeller *et al.*, 2018 ▸; Zaccai, 2011 ▸; Kneller, 2018 ▸, 2000 ▸; Zorn, 2009 ▸). Here, we analyze the ensemble-averaged single-particle self-correlation in terms of the van Hove picture (van Hove, 1954 ▸; Vineyard, 1958 ▸), as has been established and successfully employed in many other studies (Caronna *et al.*, 2005 ▸; König *et al.*, 1992 ▸; Zaccai, 2000 ▸; Doster *et al.*, 1989 ▸; Zaccai *et al.*, 2000 ▸; Doster *et al.*, 2013 ▸; Yi *et al.*, 2012 ▸; Magazù *et al.*, 2008 ▸). Being an inherently intensity-limited technique (Eckold *et al.*, 2010 ▸), full NBS spectra with a quasi-continuous energy transfer ℏ∥ω∥ ≤ 30 µeV require typical recording times of 2 to 6 h even from concentrated protein solutions, *c*_p_ ≃ 100 mg ml^−1^. In addition, calibration data such as the solvent and the sample container have to be collected with comparable quality. However, the interest in kinetically changing samples that evolve on timescales much shorter than the above recording times has increased recently. These samples can depend on external triggers or control parameters such as time (Beck *et al.*, 2019 ▸), temperature (Busch *et al.*, 2020 ▸; Guégan *et al.*, 2007 ▸; Frick *et al.*, 2013 ▸; Noferini *et al.*, 2018 ▸; Grimaldo *et al.*, 2015*a* ▸; Matsarskaia *et al.*, 2020 ▸; Di Bari *et al.*, 2023 ▸), pressure (Al-Ayoubi *et al.*, 2019 ▸), illumination (Stadler *et al.*, 2016 ▸; Stadler *et al.*, 2019 ▸), chemical potential (Grimaldo *et al.*, 2015*b* ▸) or phase transitions (Bramham & Golovanov, 2022 ▸).

New technical developments for NBS, leading to higher signal-to-noise ratios up to 1:40 000 (Appel & Frick, 2017 ▸), and new neutron guides and focusing options (Bordallo *et al.*, 2008 ▸) and phase-space transformers (Schelten & Alefeld, 1984 ▸; Hennig *et al.*, 2011 ▸) increasing the neutron flux on the sample position have already reduced the exposure time. Changes in the short-time dynamics of crystallizing proteins on a kinetic timescale of 15 min using a floating average analysis method of full QENS spectra (Beck *et al.*, 2019 ▸) have been reported recently. However, floating averages smear out events that occur on a kinetic timescale lower than the exposure time for one spectrum. Thus, to obtain good time-resolved data, high-intensity measurements with good statistics are needed on shorter timescales, or stroboscopic measure­ments can be performed (Pieper *et al.*, 2008 ▸).

The count rate for specific energy transfers can be significantly increased by choosing a displacement profile of the monochromator which selects predominately only the specified energy transfer. The acquisition time for these discrete energy transfers is significantly shorter than the acquisition time for a full QENS spectrum with a quasi-continuous energy range and has a significantly higher count rate and therefore better statistics at the given energy transfer (see also the supporting information). These measurements probing only a set of fixed energy transfers are called fixed window scan(s) (FWS). A specific case is that of zero offset (see below).

**Figure 2:**
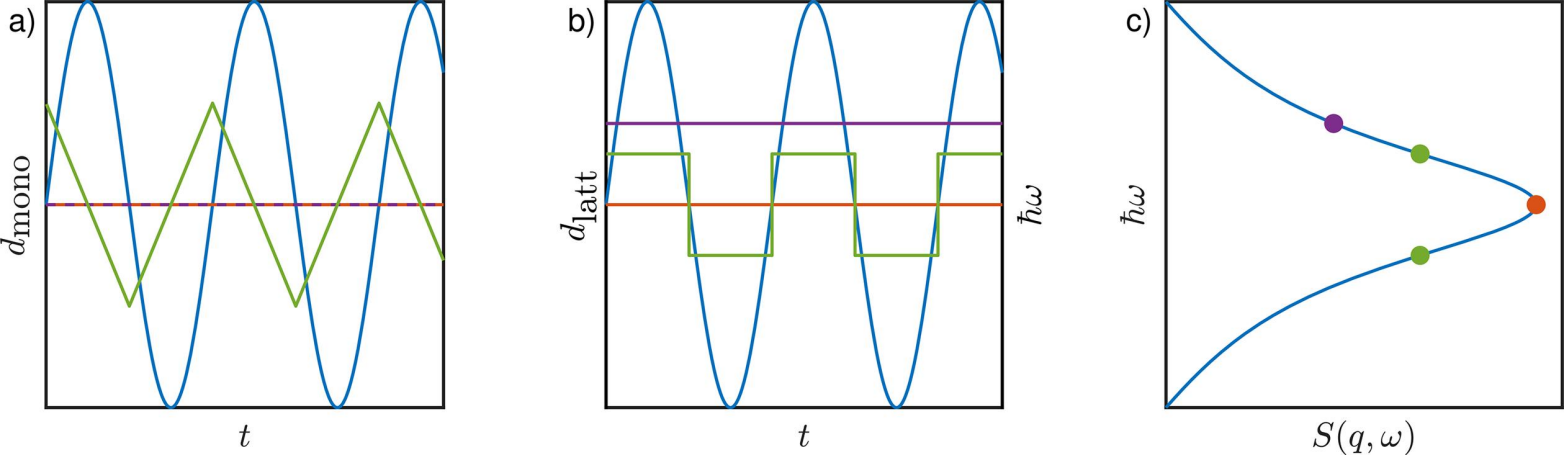
Different operation modes for backscattering spectrometers using a movable monochromator crystal. Blue, violet, green and orange lines represent the operation mode with a sinusoidal velocity profile, a stationary heated monochromator crystal, and IFWS and EFWS, respectively. (*a*) The displacement profiles *d*_mono_ translating the reference frame as a function of time. These translate to (*b*) the effective lattice spacing *d*_latt_ in the laboratory rest frame and thus to (*c*) the energy transfer ℏω encountered by the Bragg reflected neutrons. Identical monochromator and analyzer crystals, with the same crystal cut, are assumed. The detected scattering signal for the scenarios is shown in panel (*c*) in a rotated plot to have the energy transfer aligned with the plot in panel (*b*).

Different options exist to observe fixed non-zero energy transfers by what are termed inelastic fixed window scan(s) (IFWS). First, the energy may be offset by monochromator crystals with a lattice spacing different from that of the analyzers by either offsetting their temperature or using a different crystal or a different crystal cut. In Fig. 2 ▸, this first option, which is used *e.g.* on beamline IN13 at the ILL (Natali *et al.*, 2004 ▸; Natali Francesca *et al.*, 2008 ▸), is displayed with violet lines and points. Second, the velocity profile of the monochromator crystal can be chosen such that during a certain time the monochromatic neutrons have a constant offset ℏω relative to the energy set by the analyzers (Frick, 2006 ▸), due to the Doppler effect.

With a Doppler monochromator (Frick *et al.*, 2006 ▸), acquisition can be efficiently performed at fixed transfers up to ℏω ≤ 10 µeV (Frick *et al.*, 2012 ▸) within exposure times 30 < *t* < 600 s. For these FWS, the displacement profile [Fig. 2 ▸(*a*)] of the monochromator crystal is set such that it travels a major part of the available path length, typically −75 ≤ *d*_mono_ ≤ 75 mm, with a constant speed *v*, resulting in a constant effective lattice spacing *d*_latt_ in the moving reference frame [Fig. 2 ▸(*b*)] and thus in a constant neutron energy transfer [Fig. 2 ▸(*c*)]. This displacement profile corresponds to a quasi-zigzag motion, limited by the requirement of a differentiable motion obeying the technical specification of the Doppler drive (green lines and symbols in Fig. 2 ▸), allowing measurement of the energy transfers ±ℏ∥ω∥ while maintaining the good energy resolution of exact backscattering.

For identical monochromator and analyzer crystals with the same crystal cut and temperature, *v* = 0 results in ℏω = 0 (orange lines in Fig. 2 ▸) and the measurement is denoted an elastic fixed window scan(s) (EFWS). This specific case has been successfully employed in early work by Frick, Doster, Zaccai and others (Frick *et al.*, 1988 ▸; Doster *et al.*, 1989 ▸; Zaccai, 2000 ▸; Doster, 2008 ▸; Zeller *et al.*, 2018 ▸). In cases where the sample scatters mainly incoherently, this situation is also called elastic incoherent neutron scattering (EINS). Obviously, FWS cannot provide the same amount of information as full QENS measurements, which cover a quasi-continuous energy transfer range. Nevertheless, depending on the sample and kinetics, FWS are faster and may be the preferred technique. To establish a broad range of sample parameters, proteins with different sizes, namely bovine serum albumin (BSA), polyclonal immunoglobulin (Ig) and myoglobulin (Myo), were investigated at different protein concentrations and temperatures in solution (Table 1 ▸). All protein solutions were prepared by dissolving a given protein mass *m* in a volume *V* of D_2_O without further purification, resulting in the nominal protein concentration *c*_p_ := *m*/*V*. The protein solutions have been investigated previously with QENS (Doster *et al.*, 1989 ▸; Grimaldo *et al.*, 2014 ▸, 2019 ▸; Matsarskaia *et al.*, 2020 ▸; Roosen-Runge *et al.*, 2011 ▸; Girelli *et al.*, 2021 ▸). Hydrated protein powders were also measured (Table 2 ▸). Protein powders were prepared with final hydration levels of *h* = 0.29 g g^−1^ and *h* = 0.32 g g^−1^ for BSA in H_2_O and D_2_O, respectively. Given the high hydrogen ^1^H contents in the proteins, the deuterated solvents and the investigated *q* ranges, the measured signal is dominated by incoherent scattering. Different spectrometers allow investigation of the influence of different ranges in ℏ*q* and ℏω and of energy resolutions.

## 2. Contributions to the incoherent scattering signal

To describe a recorded QENS spectrum arising from diffusive dynamics, a sum of Lorentzian functions 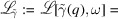

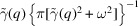
 with different width functions 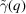 is generally employed in a suitable model function *S*(*q*, ω) (Grimaldo *et al.*, 2019 ▸). For our test protein solution samples (Table 1 ▸), we assume (Grimaldo *et al.*, 2015*c* ▸, 2019 ▸) 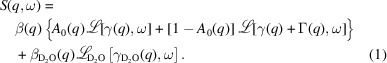

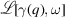
 accounts for the apparent global center-of-mass diffusion of the proteins in solution, 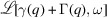 for the internal diffusive dynamics of the proteins convoluted with the global diffusion, and 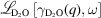 for the solvent water signal. β(*q*) and 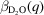 are scalars weighting the amplitudes of these contributions. *A*_0_(*q*) can be identified with the elastic incoherent structure factor (EISF) (Bee, 1988 ▸). This example model can also be applied to suspensions of soft colloids (Grimaldo *et al.*, 2019 ▸). The measured scattering signal 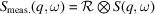 is convoluted with the spectrometer resolution function 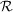 which we assume to be a zero-centered Gaussian function with an FWHM δ*E* = 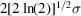 and a Gaussian standard deviation σ. For the spectrometer IN16B, we assume δ*E* = 0.9 µeV.

**Figure 3:**
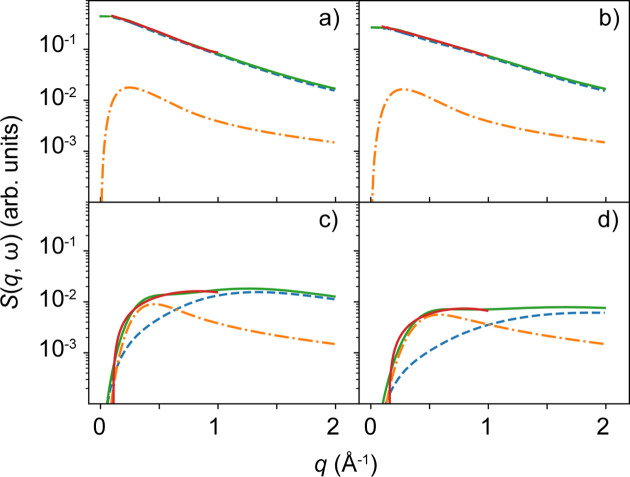
Modeled incoherent scattering functions of hypothetical soft colloids (*D* = 2 Å^2^ µeV, *D*_int_ = 35 Å^2^ µeV and τ = 0.001 µeV^−1^) in liquid suspension as a function of the momentum transfer ℏ*q* for different energy transfers ℏω, (*a*) 0 µeV, (*b*) 0.9 µeV, (*c*) 5 µeV, (*d*) 10 µeV, versus *q*. Besides the total incoherent scattering function (solid green line), the different contributions from the apparent global diffusion (blue dashed line) and from the internal diffusion (orange dashed–dotted line) are shown for several energy transfers. The red lines represent the fits to equation (2). For detailed information on the modeling see the main text and supporting information.

The *q* dependence of the widths γ(*q*) and Γ(*q*) can be described by a Fickian diffusion process (Fick, 1855 ▸) γ(*q*) = *Dq*^2^ and a jump diffusion process Γ(*q*) = 
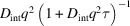
with an apparent global diffusion co­efficient *D*, an internal diffusion co­efficient *D*_int_ and a residence time τ between diffusive jumps (Singwi & Sjölander, 1960 ▸). Fig. 3 ▸ depicts experimental data and the modeled incoherent scattering depending on *q* for different ℏω. The parameterization of the EISF, the assumed quantities and representations of *S*(*q*, ω) versus ℏω for different *q* are shown in the supporting information.

We note that, in solution, proteins are subject to both translational and rotational diffusion. In numerous studies, these two contributions have been shown to be combined into an apparent global diffusion as an observable quantity, due to the large measured ℏ*q* (Grimaldo *et al.*, 2015*c* ▸), which is fully accounted for by 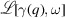 in equation (1).

Several features of the incoherent scattering function can be observed in the subplots of Fig. 3 ▸. While at low energy transfers the incoherent scattering signal decreases monotonically, it displays a maximum at higher energy transfers. In addition, the internal dynamics contribute significantly at higher energy transfers. At large *q*, the incoherent scattering function is dominated by the apparent global diffusion for all energy transfers ℏω investigated.

## 3. Generalized mean squared displacements

EINS measurements can be approximated by a model-free approach using a cumulant expansion giving access to the mean squared displacement (MSD) 〈*u*^2^〉 (Zeller *et al.*, 2018 ▸; Yi *et al.*, 2012 ▸; Rahman *et al.*, 1962 ▸; Becker & Smith, 2003 ▸), 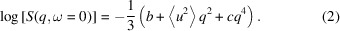
The underlying idea is to expand the Gaussian approximation, which would only have the first two contributions and therefore cover a larger *q* range. The approach in equation (2) has the advantage of being easily implemented and model free.

Applying equation (2) to IFWS results in ℏω-dependent fits such as the generalized MSD 〈*u*^2^〉_ω_ (Roosen-Runge & Seydel, 2015 ▸). The dependence on ℏω contains information on the underlying diffusive process.

This generalized MSD 〈*u*^2^〉_ω_ decays with increasing energy transfers, 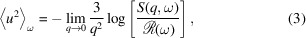
with 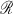 being the resolution function.

The energy dependence of 〈*u*^2^〉_ω_ contains information on the type of the diffusive process. Importantly, 〈*u*^2^〉_ω_ can result in positive and negative values, which can hint at confinement effects and free or driven motions for ℏω ≃ (2^1/2^)δ*E*, respectively (Roosen-Runge & Seydel, 2015 ▸).

**Figure 4:**
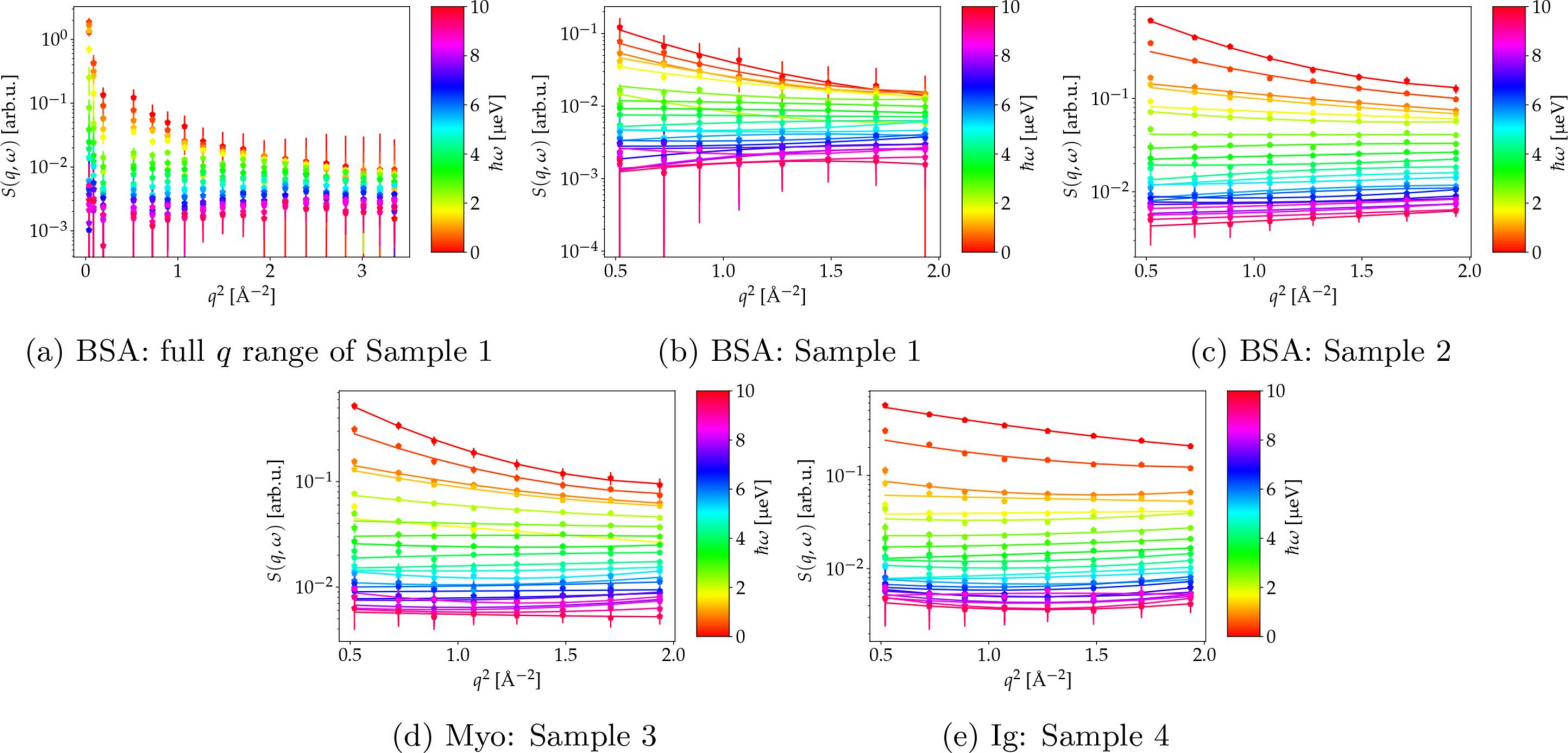
Incoherent scattering data (symbols) from protein solutions for different energy transfers ℏω (color coded) versus the scattering vector magnitude *q*, and fits by equation (2) (lines). The sample compositions are given in Table 1 ▸.

In Fig. 4 ▸, EFWS data and fits to equation (2) are shown for solution Samples 1 to 4. The fits to the modeled data from Section 2 are displayed in Fig. 3 ▸. Fig. 4 ▸(*a*) illustrates that the incoherent scattering signal cannot be described over the entire *q* range by a monotonic function [equation (2)]. At low *q*, the deviation can be explained by the slightly broader resolution function δ*E*, possible coherent scattering contributions or multiple scattering. Thus, we choose to describe *S*(*q*, ω) by equation (2) only within 0.5 < *q*^2^ < 2 Å^−2^. Figs. 4 ▸(*b*)–4 ▸(*e*) show the resulting fits for the different samples.

**Figure 5:**
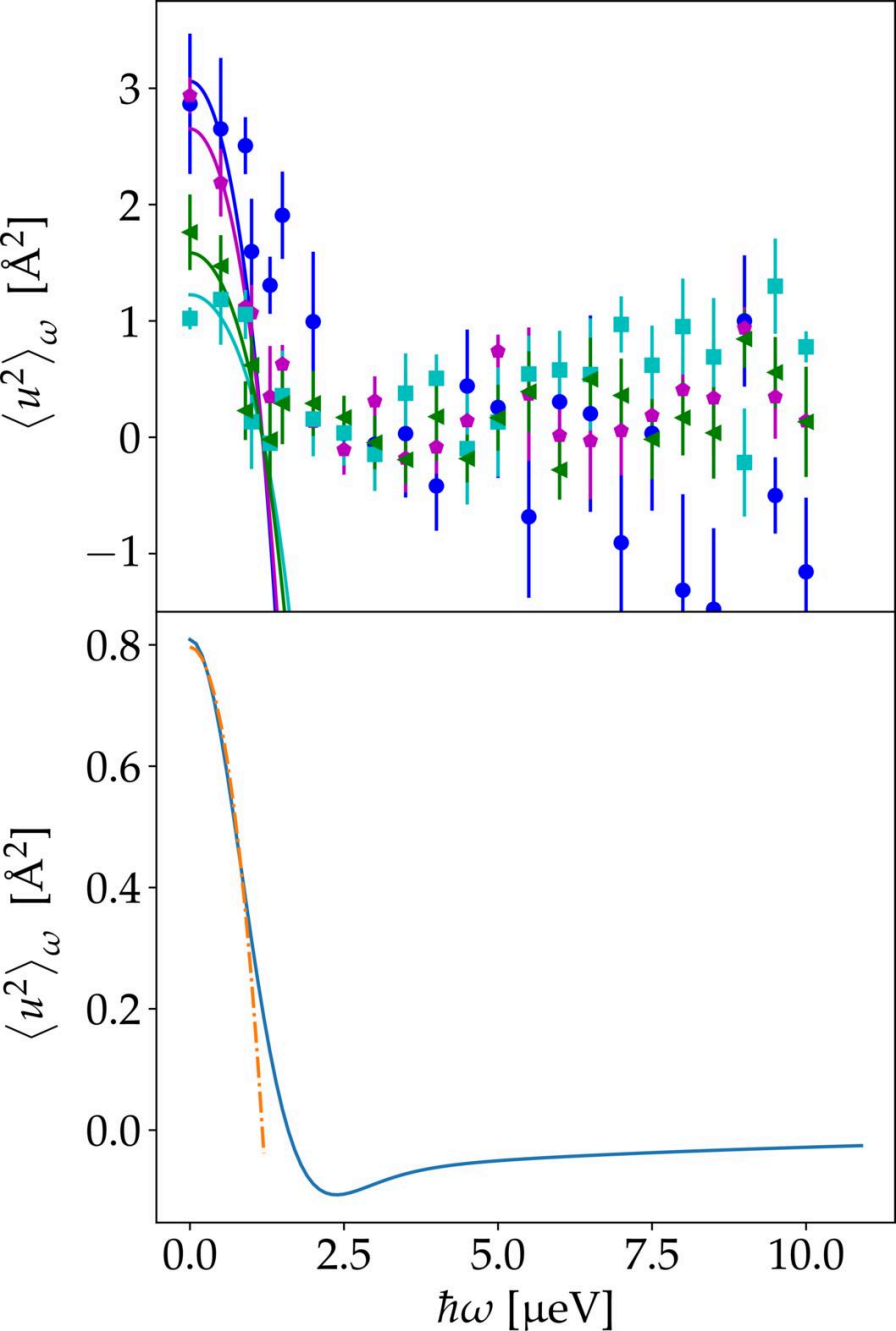
Values of 〈*u*^2^〉_ω_ obtained from the fits shown in Fig. 4 versus ℏω. Fits to equation (4) are shown as solid lines. The results are given in Table 3 ▸. (Top) Experimental results of Samples 1–4 (Table 1 ▸) at 280 K. Blue circles, green triangles, magenta pentagons and cyan squares represent values for BSA 100 mg ml^−1^ (Sample 1), BSA 500 mg ml^−1^ (Sample 2), Myo (Sample 3) and IgG (Sample 4), respectively. (Bottom) A plot of 〈*u*^2^〉_ω_ versus energy transfer for the modeled *S*(*q*, ω) from Section 2 as a blue line. The orange dashed–dotted line represents a fit to equation (4).

The extracted 〈*u*^2^〉_ω_ is plotted versus ℏω for the different samples investigated in Fig. 5 ▸. For all solution samples measured, 〈*u*^2^〉_ω_ first decays with increasing ℏω before reaching a plateau. Assuming a Fickian diffusion process with a diffusion coefficient *D*, the energy dependence can be described by (Roosen-Runge & Seydel, 2015 ▸) 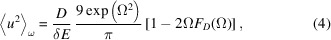
with 
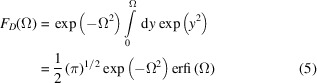
and 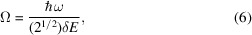
erfi(*x*) being the imaginary error function. Since at large ℏω the incoherent scattering is dominated by the internal dynamics as discussed in Section 2, deviations from the dependence in equation (4) are expected at higher ℏω and explain the plateau observed in Fig. 5 ▸. The dependence of 〈*u*^2^〉_ω_ on ℏω is therefore only fitted for lower ℏω < 1.3 µeV with equation (4) by fixing δ*E* = 0.9 µeV. These fits are shown in Fig. 5 ▸ and the associated diffusion coefficients in Table 3 ▸.

Combining polynomial expressions for the Voigt function (Hassani *et al.*, 2022 ▸; Saouessi *et al.*, 2019 ▸) with a coefficient comparison, a description of the energy dependence of 〈*u*^2^〉_ω_ might be possible and allows further investigation of the internal dynamics. However, this approach requires a parameterization of the EISF and of the internal dynamics and will therefore depend on the system. Here we evaluate the parameterized model to evaluate the effect of the internal dynamics (Fig. 5 ▸, bottom, solid line).

## 4. Analysis of FWS as sparse QENS signals

The previous sections have focused on the evaluation of the *q* dependence of one single FWS. However, similarly to the analysis of full QENS spectra, the collected data can be analyzed by taking both the energy and momentum transfer into account. The knowledge and assumptions from the total QENS fits can also be used for the data analysis. The use of several FWS at different ℏω increases the number of independent sampling points. By doing so, either a model-free analysis or more complex models accessing more parameters can be used to describe the data. This section will show initial approaches of such sparse QENS fits based on FWS, which are recombined to give QENS spectra with a very limited number of energy transfers.

To evaluate the FWS, the knowledge obtained from full QENS measurements can be used to construct a fit function. FWS performed sequentially, *i.e.* kinetically to investigate kinetically changing samples (Beck *et al.*, 2019 ▸; Pounot *et al.*, 2022 ▸, 2020 ▸), can be grouped into a sparse QENS spectrum and can then be analyzed similarly to the QENS spectra.

While the polynomial approach to analyzing FWS presented in the preceding Section 3 is model free, the approach presented here relies on modeling existing knowledge from full QENS. On the one hand, on the basis of the Nyquist–Shannon sampling theorem (Shannon, 1998 ▸), this can allow observation of faster kinetic changes in the system, *e.g.* on a timescale of one minute instead of several hours. On the other hand, it might lead to systematic errors if the system changes in a way inconsistent with the model employed. Here, a different number of Voigt functions 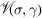 are used to describe the experimental data. The parameter σ is fixed according to the resolution function and the scaling parameter and γ are kept free as fit parameters.

**Figure 6:**
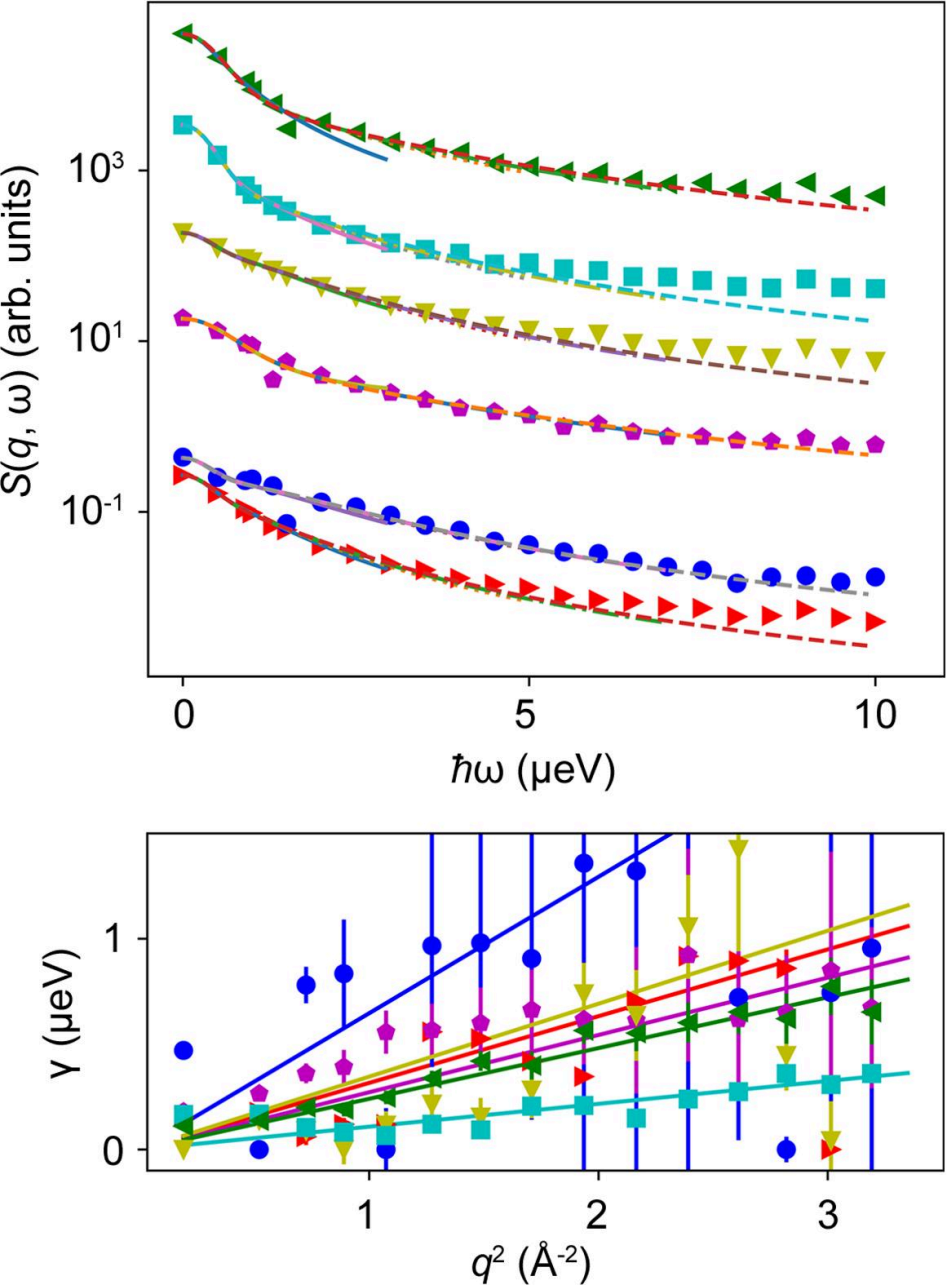
(Top) Sparse QENS analysis for different samples using two Voigt functions to fit the energy dependence. A significantly better agreement is observed for high energy transfers, where different diffusive processes contribute substantially to the scattering signal. Fits are shown at *q* = 1 Å^−1^ for different maximum energy transfers ℏω_max_ = 3 µeV (solid blue line), ℏω_max_ = 5 µeV (orange dotted line), ℏω_max_ = 7 µeV (green dashed–dotted line) and ℏω_max_ = 10 µeV (brown dashed line). The different curves are shifted by a factor of ten each for better visibility. (Bottom) The *q*^2^ dependence of the corresponding widths of the fit for ℏω < 10 µeV and a fit of γ = *Dq*^2^ to determine the diffusion coefficient *D*. In both plots, blue spheres, magenta pentagons, cyan squares, olive down pointing triangles, red right pointing triangles and green left pointing triangles represent values for BSA 100 mg ml^−1^ at 280 K, myoglobin 500 mg ml^−1^ at 280 K, polyclonal IgG 500 mg ml^−1^ at 280 K, BSA 500 mg ml^−1^ at 310 K, BSA 500 mg ml^−1^ at 295 K and BSA 500 mg ml^−1^ at 280 K, respectively.

Depending on the number of fit parameters and the energy transfers available, either fits can be performed similarly to the classical QENS analysis for each momentum transfer separately or they can be performed for all energy and momentum transfers simultaneously. Fits of the different spectra with one single Voigt function (Fig. S7) show a reasonably good agreement for low energy transfers, but larger deviations can be observed at higher energy transfers. To describe the energy dependence more adequately, a second Voigt function is added to the model. The fit results are shown in Fig. 6 ▸. While most samples show similar fit functions independent of the cutoff Δ*E*, this agreement clearly depends on the sample dynamics, as expected. It becomes clear that, depending on the hierarchical diffusive processes observable on the length and timescales of the instruments, a different number of energy transfers are necessary to capture all processes. A disentanglement of all contributions is not always straightforward.

## 5. Extracting effective diffusion coefficients from two FWS at different energy transfers

**Figure 7:**
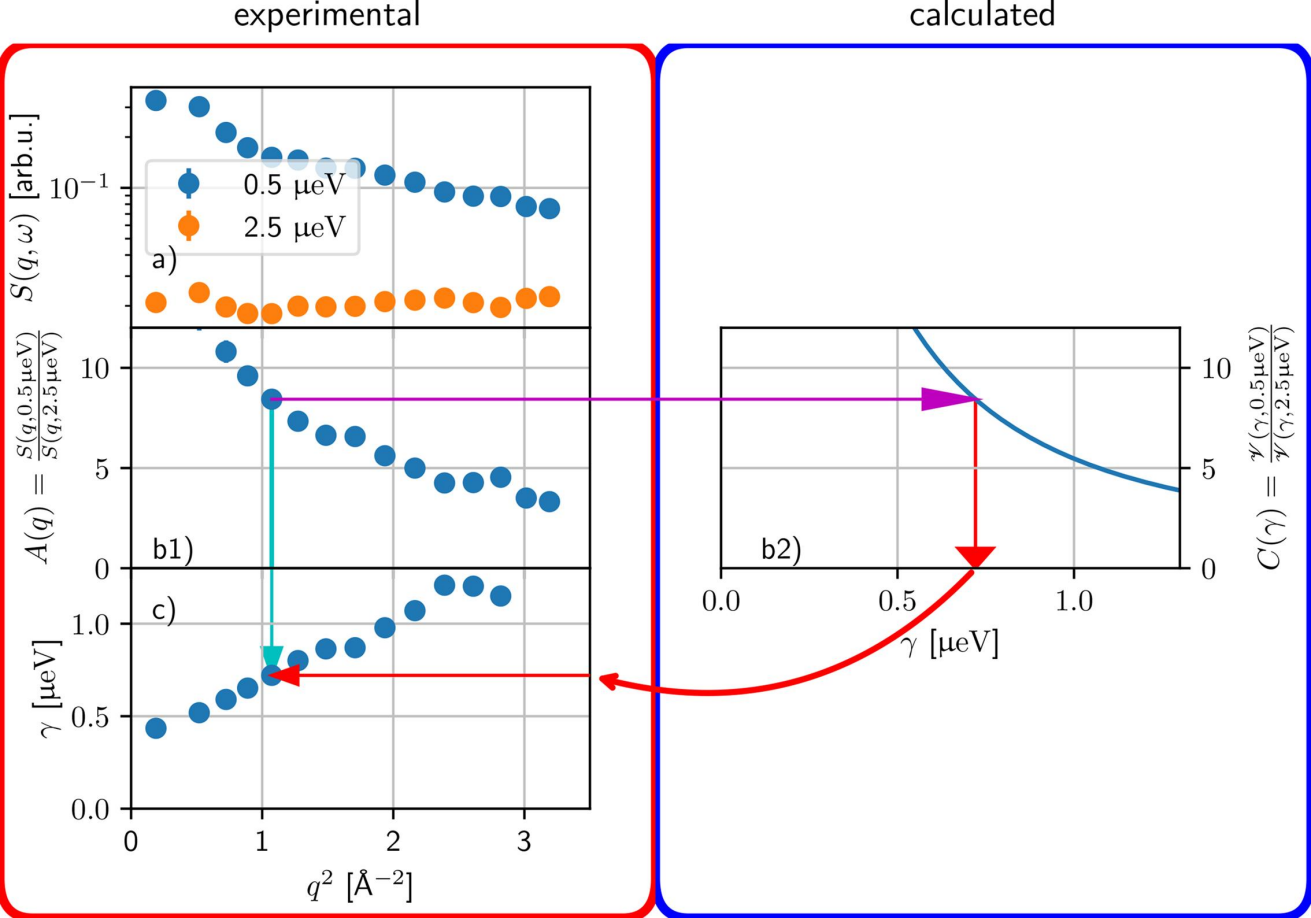
A graphical illustration of the data analysis path explained in Section 5. For illustration, data from polyclonal Ig (Sample 4, Table 1 ▸) have been used.

The most sparse FWS acquisition protocol would be the collection of only two FWS. Here, we address this case by analyzing the FWS in a way motivated by the QENS analysis but still using a model-free approach. The only assumption within this framework is that the observed broadening of the scattering signal can be described by a Lorentzian function which dominates the scattering signal within the energy transfer investigated. In principle, other line shapes can also be applied. Similar to the analysis of full NBS spectra, this analysis offers a *q*-dependent half-width at half-maximum (HWHM). Several steps, illustrated in Fig. 7 ▸, are necessary for the analysis:

(i) For both energy transfers, the empty sample container contribution is subtracted from the FWS. Subsequently, the ratio 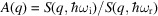 between these two FWS is determined [Fig. 7 ▸(*b*1)].

(ii) To obtain the HWHM as a function of *q*, a calibration curve *C*(γ) can be calculated [Fig. 7 ▸(*b*2)], 
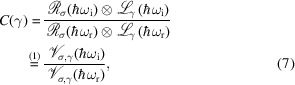
with the energy transfers ℏω_i_ and ℏω_r_ of both FWS (subscript indices r and i refer to different energy offsets). In step (1), a centered Gaussian function as resolution function is assumed, but more complicated expressions for 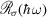 are possible in numerical evaluation.

(iii) The calibration curve *C*(γ) is used to convert the experimental ratio value *A*(*q*) into the line broadening γ = *C*^−1^[*A*(*q*)] [Fig. 7 ▸(*c*)].

(iv) The *q* dependence of γ can then be analyzed with known models.

**Figure 8:**
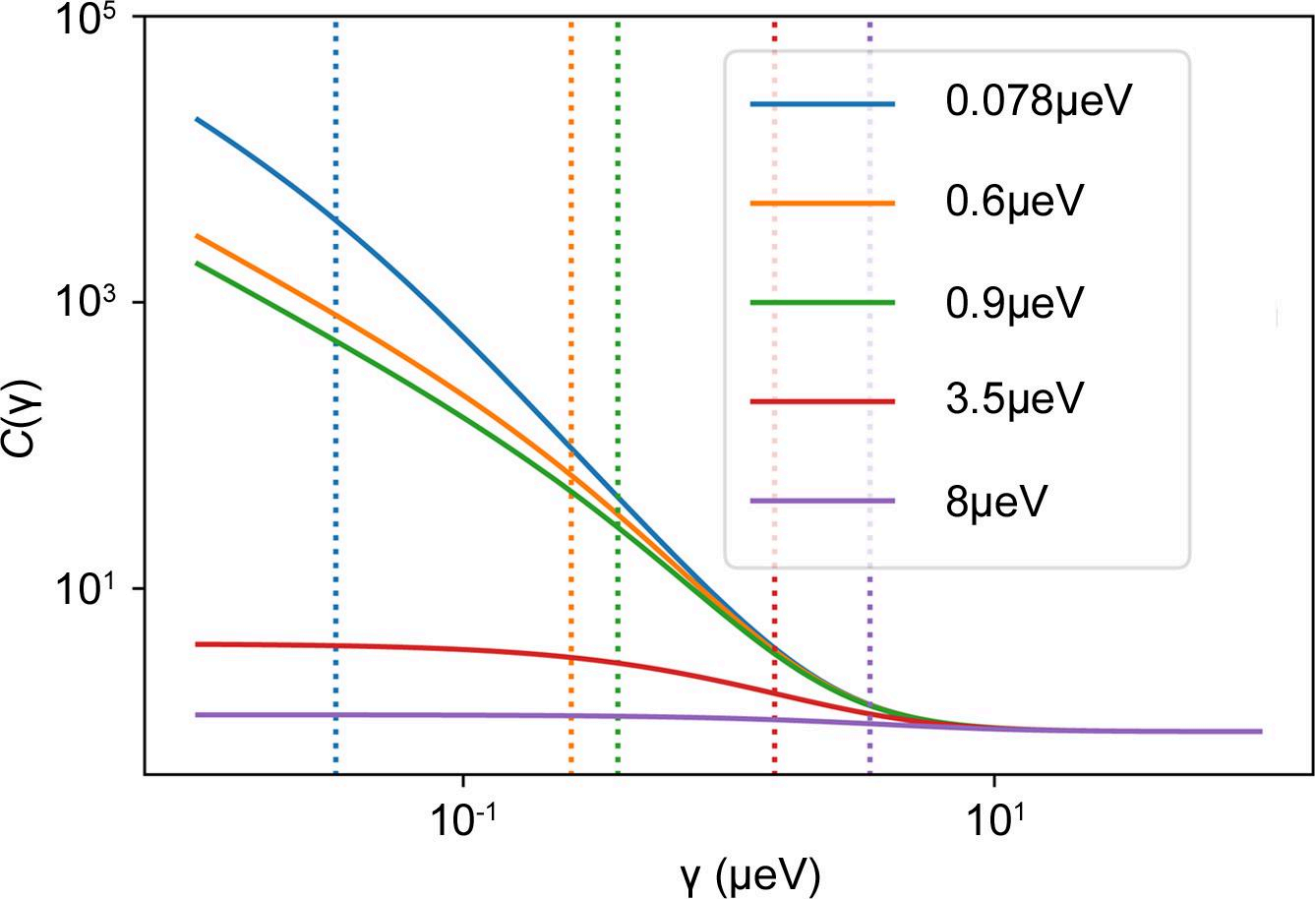
Resolution-dependent calibration curves *C*(γ) based on equation (7) calculated with ℏω_i_ = 0 µeV and ℏω_r_ = 2.5 µeV. Different assumed instrument resolutions are color coded and the vertical dashed lines represent the assumed resolution for calculation.

In Fig. 8 ▸, *C*(γ) is plotted versus γ for different resolutions with FWHM δ*E*. The dependence of *C*(γ) on different energy transfers chosen for the FWS is shown in the supporting information. The FWHMs were chosen to match the resolutions of backscattering spectrometers such as the GaAs prototype on IN16B [δ*E* = 0.078 µeV (Kuhlmann *et al.*, 2019 ▸)], the spectrometer on SPHERES [δ*E* = 0.6 µeV (Wuttke *et al.*, 2012 ▸)], the unpolished Si(111) versions on IN16B (Kuhlmann *et al.*, 2019 ▸), HFBS (Meyer *et al.*, 2003 ▸) and EMU (Souza *et al.*, 2016 ▸) (δ*E* = 0.9 µeV), the IN16B BATS option [δ*E* = 3.5 µeV (Mamontov & Herwig, 2011 ▸; Appel *et al.*, 2018 ▸; Beck *et al.*, 2019 ▸)] and the spectrometer on IN13 [δ*E* = 8 µeV (Natali *et al.*, 2004 ▸; Natali Francesca *et al.*, 2008 ▸)]. In practice, the resolution is not independent of *q*. Therefore, in general, a *q*-dependent calibration curve *C*(γ, *q*) has to be used. This approach has several restrictions concerning the observable global dynamics. If the tracer particle moves too slowly, the ratio observed is dominated by the resolution function and would suggest an immobile particle scattering only elastically. This situation is observed for instance in the case of crystallizing samples (Beck *et al.*, 2019 ▸). As can be seen in Fig. 8 ▸, the calibration curves level off slightly at low γ. If an experimental ratio with its corresponding errors close to this value were translated into the corresponding γ, even a small error in the ratio would lead to a large error in γ.

However, in cases where the dynamics are too fast, the chosen energy offsets do not differ significantly and the ratio is close to unity. Additionally in this case, γ cannot be determined unambiguously anymore. To investigate the influence of the resolution parameterization on the obtained values of γ, the *q*-dependent description of the resolution function of IN16B, using two free Gaussian functions, has been applied, yielding similar results to the analysis using only one single Gaussian function (see Fig. S9 in the supporting information).

Looking at γ(*q*), an offset γ(*q* → 0) > 0 can be observed. Therefore, we allow for an offset γ_0_ in the fitting and determine *D* from the *q* dependence as usual, *i.e.* we fit the data to 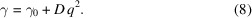


Experimentally, the scattering signal is rarely described by a single diffusive process, since different contributions, such as different hierarchically superimposed diffusive processes and scattering from the solvent, are present, which might explain the offset γ_0_.

Besides the resolution, the energy offset of the FWS also influences the calibration curve and has to be chosen adequately. When several hierarchical levels of diffusive processes are present, the approximation by a single Lorentzian function may no longer be valid at large ℏω. In addition, the solvent becomes more dominant at larger ℏω. To ensure that mainly the global dynamics are probed and immobile scatterers do not contribute, one is interested in measuring at small ℏω > 0, where the global dynamics dominate the QENS signal. Depending on the system studied, it is therefore important to choose the optimal energy offsets and a suitable instrument, which determines the resolution function.

**Figure 9:**
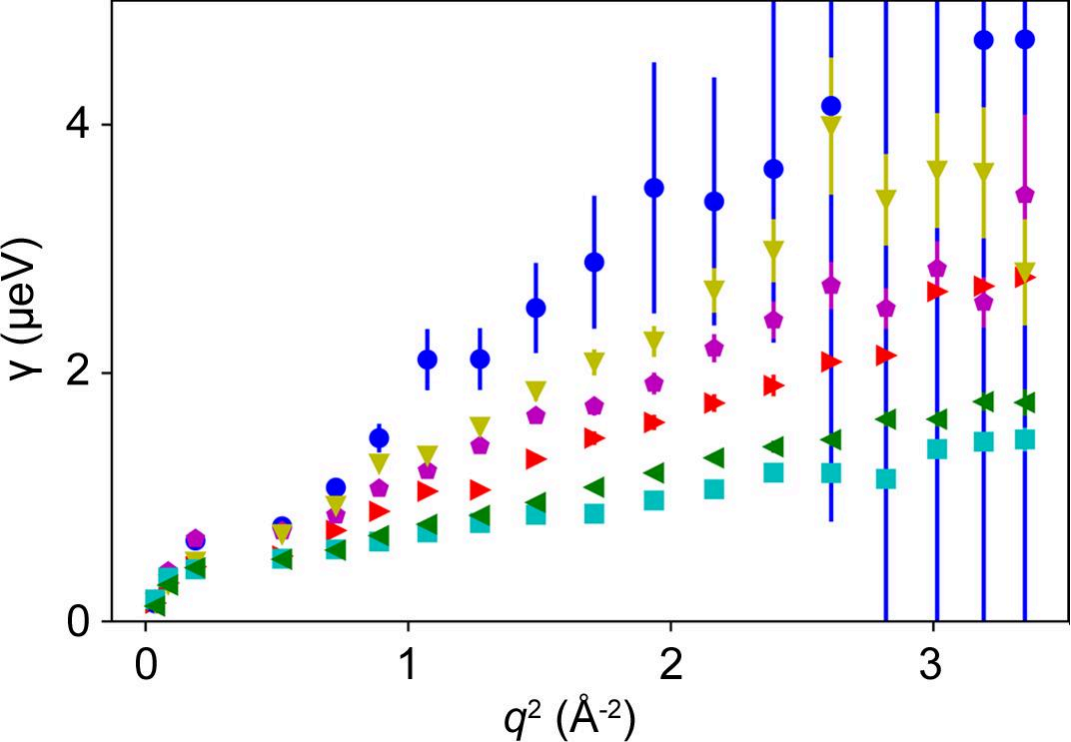
Plots of γ versus *q*^2^ derived from FWS collected at ℏω_i_ = 0.5 µeV and ℏω_r_ = 2.5 µeV for the samples investigated. Blue spheres, magenta pentagons, cyan squares, olive down pointing triangles, red right pointing triangles and green left pointing triangles represent values for BSA 100 mg ml^−1^ at 280 K, myoglobin 500 mg ml^−1^ at 280 K, polyclonal IgG 500 mg ml^−1^ at 280 K, BSA 500 mg ml^−1^ at 310 K, BSA 500 mg ml^−1^ at 295 K and BSA 500 mg ml^−1^ at 280 K, respectively.

**Figure 10:**
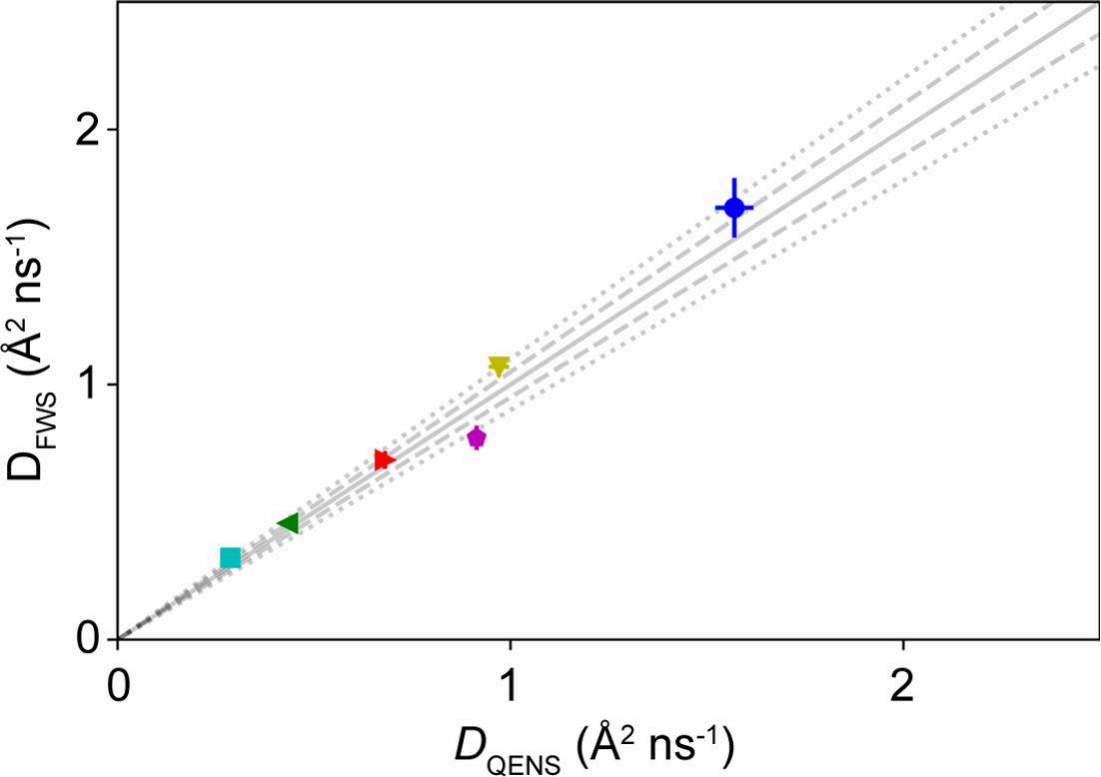
A comparison of the diffusion coefficients obtained from the FWS with those from the full QENS spectrum analysis. The different colored symbols represent the samples listed in Table 1 ▸. The solid, dashed and dotted lines represent a perfect agreement and a 5% and 10% deviation, respectively.

**Figure 11:**
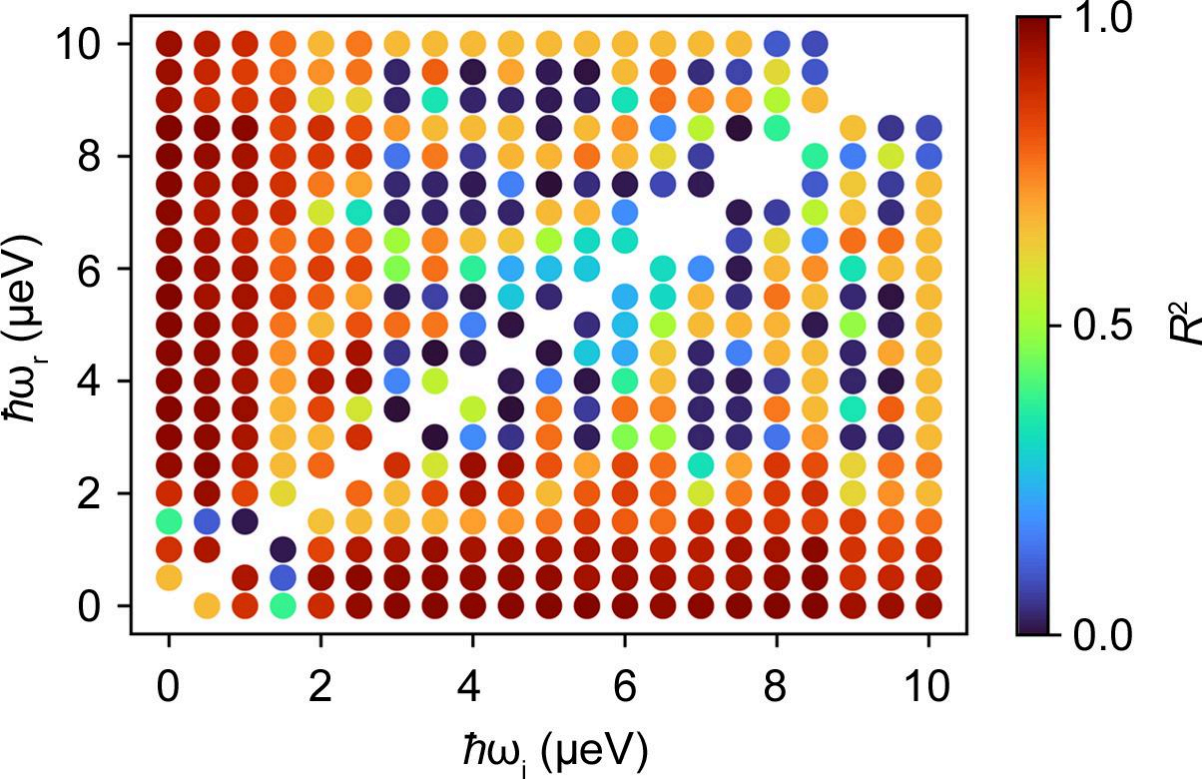
The coefficient of determination of the results obtained from the QENS analysis and the ratio analysis as a function of the chosen energy transfers ℏω_i_ and ℏω_r_. A reasonably good agreement can be observed for cases where one of the energy transfers is below and the other above ℏω = 1.5 µeV

To obtain suitable offsets which result in sample-independent results, we systematically tested different combinations of ℏω_i_ and ℏω_r_ on IN16B with Si(111) crystals (δ*E* ≃ 0.9 µeV). In Fig. 9 ▸, γ is displayed versus *q*^2^ for the different samples investigated using the energy transfers ℏω_i_ = 2.5 µeV and ℏω_r_ = 0.5 µeV. In Fig. 10 ▸, the diffusion coefficients thus obtained are plotted versus the diffusion coefficients determined from the full QENS spectrum analysis. A reasonable agreement of the two methods is observed, with a coefficient of determination *R*^2^ = 0.97. In Fig. 11 ▸, *R*^2^ is displayed for all combinations of energy transfers investigated. It shows that for a good agreement with the results from full QENS spectra, one energy transfer should be below ℏω ≲ 1.5 µeV while the other energy transfer should stay above this threshold. Outside this observed energy transfer range, the *q* dependence of γ is characterized for many ℏω combinations by a *q*-independent plateau (Fig. S8), indicating the limits of the proposed framework.

The energy transfers have been found to be reliable for our example protein solutions. For samples with significantly different features in the QENS signal, different optimal energy transfers may apply for their investigation, *e.g.* masking out the signal of an elastic contribution. Although the choice of the energy transfers may influence the absolute value of the obtained diffusion coefficient *D*, the method can be applied to follow qualitatively the effect of parameters such as temperature (Grimaldo *et al.*, 2015*a* ▸; Matsarskaia *et al.*, 2020 ▸), pressure (Caliò *et al.*, 2022 ▸) or time (Pounot *et al.*, 2020 ▸; Beck *et al.*, 2019 ▸) on relative changes in *D*.

## 6. Applicability to hydrated powder samples

The methods developed above have mainly focused on samples in solution. The investigation of hydrated powders allows us to observe directly the internal diffusive properties of samples by suppressing both the global translational and rotational contributions and the contribution from the solvent. The *q* dependence of γ can differ from the Fickian diffusion (Fick, 1855 ▸) often present in colloidal suspensions and can be described by different models (Singwi & Sjölander, 1960 ▸; Chudley & Elliott, 1961 ▸; Hall & Ross, 1981 ▸). Over the years, different descriptions have been developed and employed for the analysis of EFWS on different hydrated powders (Doster *et al.*, 1989 ▸; Zeller *et al.*, 2018 ▸; Kneller & Chevrot, 2012 ▸; Tokuhisa *et al.*, 2007 ▸; Becker & Smith, 2003 ▸; Yi *et al.*, 2012 ▸; Peters & Kneller, 2013 ▸; Vural *et al.*, 2015 ▸; Liu *et al.*, 2017 ▸; Matsuo & Peters, 2022 ▸).

The aim of this section is to show the applicability of the methods to powder samples hydrated with H_2_O or D_2_O. Different samples were prepared and measured with different energy transfers, as specified in Table 2 ▸. The change from D_2_O to H_2_O substantially increases the incoherent contribution of the hydration water. Differences in the obtained results are therefore a combination of possible isotope effects on the system investigated (Braun *et al.*, 2017 ▸) and the change in the relative scaling of different contributions.

**Figure 12:**
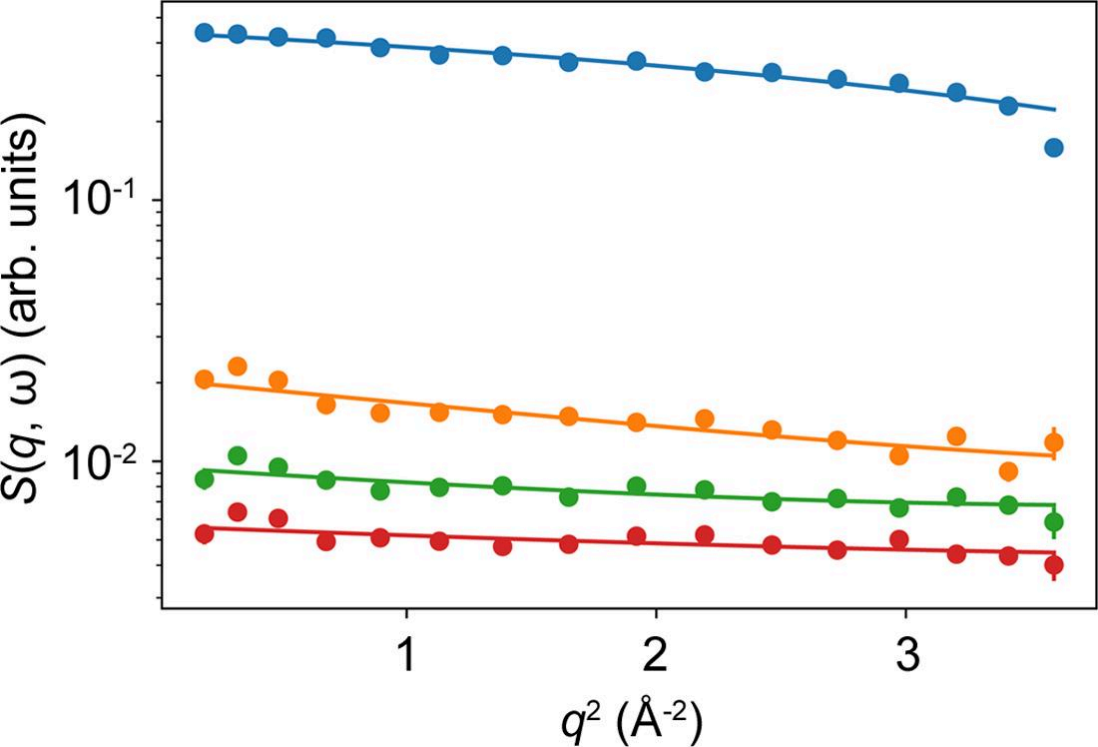
Fits of BSA powder hydrated with H_2_O (Sample 5, Table 2 ▸), measured on IN16B at *T* = 280 K, using equation (2). Blue, orange, green and red symbols represent energy transfers of ℏω = 0, 1.3, 3 and 6 µeV, respectively.

First, the energy transfers are analyzed within the framework of generalized MSD. Figs. 12 ▸ and 13 ▸ show the fits by equation (2) and the corresponding results for different energy transfers.

**Figure 14:**
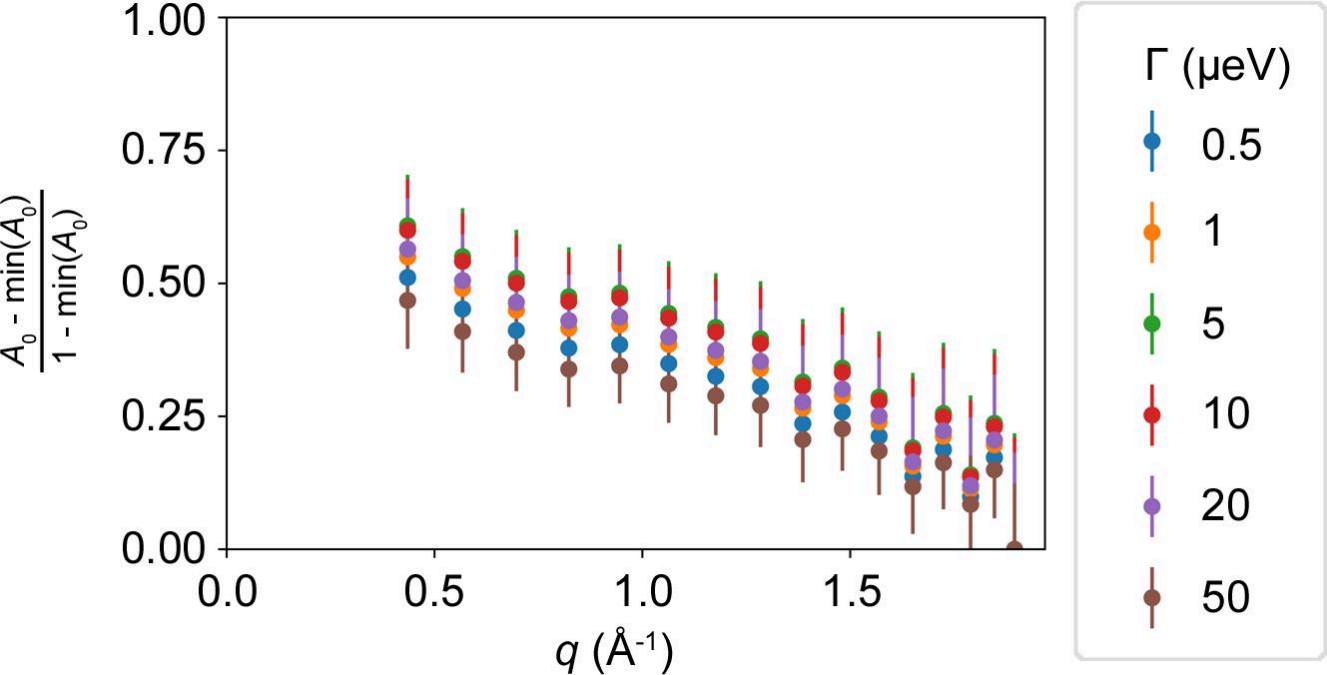
EISF corrected for the immobile fraction determined from FWS recorded from the H_2_O hydrated BSA powder. A good agreement can be observed between the results for the different choices of Γ.

Since the center-of-mass motion of the proteins, which has been investigated with the FWS ratios in the solution samples, is not present in the powder samples [*i.e.* γ = 0 in equation (1)], the found line width corresponds to the second Lorentzian Γ in equation (1). In practical terms, to exclude the elastic peak, two IFWS have to be taken to access Γ from equation (1). Moreover, the combination of IFWS with EFWS accesses information on the EISF (see the supporting information, Section S7). On the basis of the energy transfers ℏω = 3 and 6 µeV, we apply the method described in Section 5 to determine Γ(*q*). For the two samples investigated, the *q* dependence is shown in Fig. S10 for *T* = 280 K. It can be seen that the broadening determined is basically *q* independent in the investigated *q* range. By investigating the ratio between the EFWS and an IFWS, it is possible to extract the EISF. A detailed procedure is given in the supporting information. The procedure incorporates the broadening Γ, whose value mainly influences the immobile fraction of the EISF *A*_0_(*q* → ∞). Fig. 14 ▸ depicts the EISF determined for the H_2_O hydrated sample, corrected for the immobile fraction, for different values of Γ. A good agreement between the curves and with the previously obtained EISF of dissolved BSA (Grimaldo *et al.*, 2015*a* ▸) can be observed.

## 7. Influence of the instrument resolution

To investigate the influence of the instrument resolution on the ratio analysis, the BSA powder samples hydrated with H_2_O (Sample 5) and D_2_O (Sample 6) were measured on IN13 as a function of temperature with energy transfers ℏω = 3 and 9 µeV. Compared with IN16B, the backscattering spectrometer IN13 is characterized by its higher incident neutron energy (Teixeira *et al.*, 2008 ▸), resulting in a broader energy resolution δ*E* ≃ 8 µeV but also a significantly larger *q* range, 0.1 < *q* < 4.9 Å^−1^. A detailed investigation of the influence of the resolution function and the *q* range has been reported previously (Gabel, 2004 ▸).

Assuming that the elastic contribution to the spectra dominates the FWS at the lower energy transfer ℏω = 3 µeV, which is nominally within the energy resolution of the instrument, the framework to determine the EISF can be applied. The obtained values also depend on the choice of Γ (see Fig. S13). However, a direct renormalization by the immobile fraction as in the case of the EFWS fails. By applying the framework from Section 5 it is possible to determine the broadening Γ. The values of Γ observed on IN13 are comparable to those determined on IN16B with similar offsets (ℏω_i_ = 3 µeV, ℏω_r_ = 6 µeV, see Fig. S14). Analogously to the method shown in Section 5, it is therefore possible to determine the internal diffusive properties of the proteins with FWS acquired with energies which are within the energy resolution of the instrument.

## 8. Conclusions

We have developed a new analysis framework to treat quasi-elastic neutron spectroscopy data recorded at discrete energy transfers and have identified how to choose small sets of energy transfers optimally such that a quantitative agreement can be obtained with the analysis of full QENS spectra. We have demonstrated this framework for a representative range of sample conditions employing different protein solutions. The application of the framework to hydrated powders has demonstrated the suitability of the approach to investigate other systems than suspensions with the same framework. For hydrated powders, the description based on one single Lorentzian function approximating the scattering function in the energy transfer range investigated is not sufficient. Therefore, only relative changes in the obtained parameters (as a function of solute or temperature) can be investigated. To obtain physically meaningful parameters, the function describing the energy dependence of the scattering function and the used energy transfers might have to be adapted.

The concept of fixed-window scans can be generalized. Besides the use of Bragg reflections from crystals, the energy of the incoming neutrons can be defined by the time of flight of pulsed neutron beams, *i.e.* by the neutron dispersion. The energy resolution of such spectrometers is slightly broader, but significantly higher energy transfers can be accessed [BASIS (Mamontov & Herwig, 2011 ▸), IN16B in BATS mode (Appel *et al.*, 2018 ▸), TOFTOF (Unruh *et al.*, 2007 ▸), MIRACLES (Tsapatsaris *et al.*, 2016 ▸), OSIRIS (Telling *et al.*, 2005 ▸; Demmel *et al.*, 2018 ▸), IRIS (Demmel *et al.*, 2018 ▸; Campbell *et al.*, 2000 ▸), DNA (Shibata *et al.*, 2015 ▸; Seto *et al.*, 2017 ▸) and MARS (Tregenna-Piggott *et al.*, 2008 ▸)]. Fixed-window scans are thus given by a fixed time-of-flight window. While the frameworks presented here should be applicable to these scans, the generally larger energy transfers and resolutions will shift the timescales accessed. In neutron spin–echo spectroscopy as well, the concept of a fixed-window scan may be applied by measuring at a fixed Fourier time while changing a sample parameter such as the temperature (Girelli *et al.*, 2021*a* ▸; Kulda *et al.*, 2004 ▸).

In combination with the available capabilities of neutron backscattering spectrometers at high-flux neutron sources, this new framework allows a reduction in the acquisition time needed by nearly two orders of magnitude. It can therefore serve to investigate nanosecond diffusive dynamics in samples that undergo kinetic changes on timescales of minutes, or the dependencies on control parameters such as pressure, temperature or light. Thus, topics of current interest including the dynamics of protein aggregation, liquid–liquid phase separation and crystallization can be investigated. Numerous science cases may be given by systems that evolve on minute timescales. For instance, during a protein crystallization process, the dynamic equilibrium of freely diffusing proteins and of proteins bound to a crystallite may be followed (Beck *et al.*, 2019 ▸; Sauter *et al.*, 2015 ▸), which gradually shift towards the crystalline phase. Light-induced changes on the molecular level in proteins that are already being systematically investigated by experiments accessing structure (Röllen *et al.*, 2018 ▸) will become accessible to dynamic studies. For instance, in studies of light-induced conformational changes of proteins that are highly relevant *e.g.* in photosynthesis (Golub *et al.*, 2023 ▸), the gradual relaxation of the protein dynamics to the ground state may be observed. The kinetics of pathological protein aggregation may also be studied *in vitro* (Pounot *et al.*, 2020 ▸). More generally, the mobility of adsorbates on crystallites, such as organic mol­ecules on ice forming in solution (Clarke & Arnold, 2002 ▸; Joliat *et al.*, 2022 ▸), may be studied kinetically or at low concentrations, resulting in weak signals. Such adsorption–desorption kinetic phenomena on tiny crystals are important in fields ranging from the food industry (Petzold & Aguilera, 2009 ▸) to astrophysics (Boogert *et al.*, 2015 ▸), atmospheric chemistry (Solomon, 1999 ▸) and even the pipeline industry (Kashchiev & Firoozabadi, 2003 ▸). In some cases, such as for the said photo-activated proteins, the sample may be reversibly ‘cycled’. Thus, the several offsets for a set of fixed-window data may be recorded consecutively during separate cycles of the identical same sample.

The new framework might also improve the scope of neutron sources with lower neutron flux (Ott *et al.*, 2023 ▸; Brückel *et al.*, 2023 ▸). However, we point out that on pulsed neutron sources spectrometers exploiting the time structure of the source – notably via the neutron beam dispersion – to define the incident wavelength are generally preferred over spectrometers with a backscattering monochromator crystal. The fixed-window technique, in contrast, achieves its best efficiency on spectrometers equipped with a monochromator crystal to define the incident wavelength.

Further development of the technique may also allow disentangling of distinct diffusive processes by taking more than two energy transfers into account. In this article, we have mainly discussed the moving monochromator setup used for the majority of test experiments performed. However, without loss of generality, the concepts are applicable to the temperature-controlled monochromator setup (Cook *et al.*, 1992 ▸; Kuhlmann *et al.*, 2019 ▸; Natali *et al.*, 2004 ▸; Natali Francesca *et al.*, 2008 ▸), with the limitation that the temperature change is not quasi-instanteous, or to other designs of switching monochromators, *e.g.* by a set of monochromators mounted on a disc-type monochromator changer. Such concepts might be employed for instance at future medium-flux ‘compact’ neutron sources (Ott *et al.*, 2023 ▸; Brückel *et al.*, 2023 ▸).

## 9. Data availability

The neutron data are curated by the ILL and are accessible via references Beck *et al.* (2021 ▸, 2016 ▸) and Grimaldo *et al.* (2016 ▸).

## Supplementary Material

Additional theory and figures. DOI: 10.1107/S1600576724003820/jo5102sup1.pdf

Experimental data curated by the ILL: https://dx.doi.org/10.5291/ILL-DATA.1-20-69

Experimental data curated by the ILL: https://dx.doi.org/10.5291/ILL-DATA.9-13-637

Experimental data curated by the ILL: https://dx.doi.org/10.5291/ILL-DATA.9-13-628

## Figures and Tables

**Figure 13:**
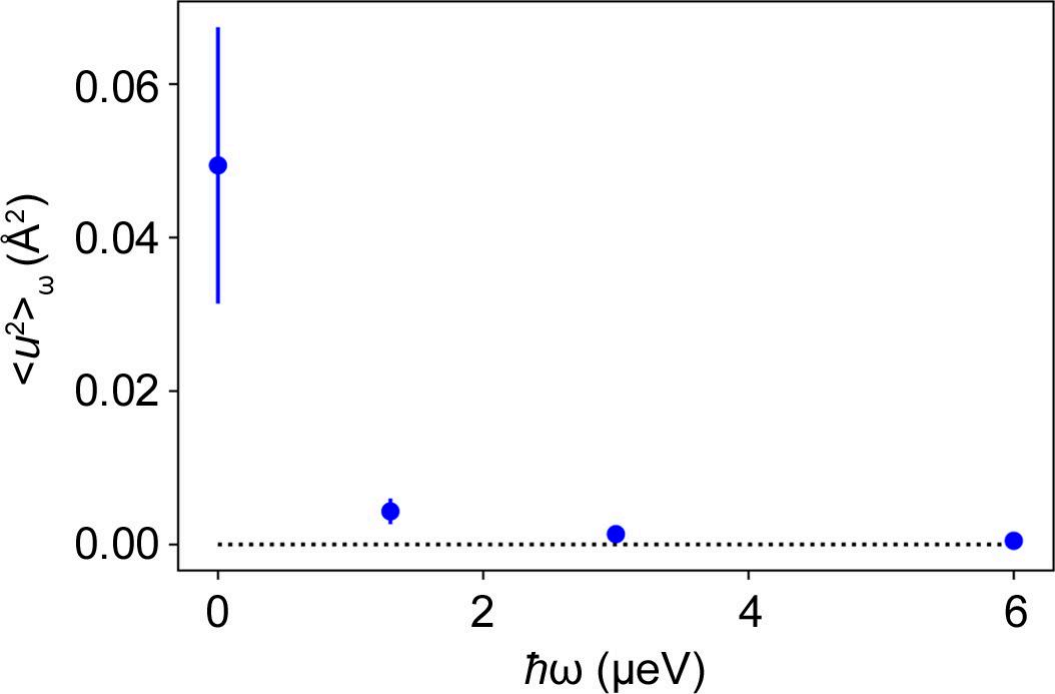
The generalized MSD of BSA powder hydrated with H_2_O (Sample 5) as a function of ℏω obtained from measurements on IN16B (Fig. 12) at *T* = 280 K.

**Table 1:** Measured liquid protein solution sample conditions All samples were measured during experiment 1-20-69 (Beck *et al.*, 2021 ▸) on IN16B at the ILL. Full QENS spectra were measured with energy resolution δ*E* = 0.9 µeV FWHM and 30 µeV dynamic range with a scattering vector range 0.1 < *q* < 1.8 Å^−1^. FWS were measured with the same energy resolution and same *q* range at energy transfers in the interval ℏω = [0, 10] µeV with a step size of 0.5 µeV.

Sample No.	Temperature (K)	Sample composition
1	280	BSA 100 mg ml^−1^
2	280, 295, 310	BSA 500 mg ml^−1^
3	280	Myo 500 mg ml^−1^
4	280	Polyclonal Ig 500 mg ml^−1^

**Table 2:** Powder sample conditions measured on the NBS instruments IN16B [beamtime 9-13-637 (Beck *et al.*, 2016 ▸), δ*E* = 0.9 µeV, 0.1 < *q* < 1.8 Å^−1^] and IN13 [9-13-628 (Grimaldo *et al.*, 2016 ▸), δ*E* = 8 µeV, 0.19 < *q* < 4.9 Å^−1^] Both samples were measured during temperature ramps.

Sample No.	Instrument	ℏω (µeV)	Sample composition
5	IN16B	0, 1.3, 3, 6	BSA hydrated with H_2_O
	IN13	3, 9	
6	IN16B	0, 1.3, 3, 6	BSA hydrated with D_2_O
	IN13	3, 9	

**Table 3:** Diffusion coefficients obtained from the fits of the energy dependence of 〈*u*^2^〉_ω_ at 280 K shown in Fig. 5 ▸

Sample No.	Sample composition	*D*_GMSD_ (Å^2^ ns^−1^)
1	BSA 100 mg ml^−1^	1.46 ± 0.41
2	BSA 500 mg ml^−1^	0.76 ± 0.11
3	Myo 500 mg ml^−1^	1.27 ± 0.19
4	Polyclonal Ig 500 mg ml^−1^	0.58 ± 0.11
	Modeled data	0.38 ± 0.01
