# Supplementary material for: Accessing self-diffusion on nanosecond time and nanometre length scales with minute kinetic resolution
Source: J Appl Crystallogr. 2024 Jun 7;57(Pt 4):912–24. doi: 10.1107/S1600576724003820 (PMC11299610; doi:10.1107/S1600576724003820)
Supplement: Supplementary file 1 [file j-57-00912-sup1.pdf]

JOURNAL OF APPLIED CRYSTALLOGRAPHY

Volume 57 (2024)

Supporting information for article:

Accessing self-diffusion on nanosecond time and nanometre length scales with minute kinetic resolution

Christian Beck, Felix Roosen-Runge, Marco Grimaldo, Dominik Zeller, Judith Peters, Frank Schreiber and Tilo Seydel

S1 Parametrization of modeled $S(q, \omega)$ data

The incoherent QENS signal of the protein is modeled using two Lorentzian functions $\mathscr{L}_{\gamma_i}(\omega)$ with a width γ_i to describe the apparent global and internal diffusion, respectively. The instrument resolution $\mathscr{R}_\sigma(\omega)$ is modeled using one Gaussian function with $\sigma = 0.9\,\mu\text{eV}$. The scattering function therefore reads:

$$S(q,\omega) = \mathscr{R}_\sigma(\omega) \otimes \{A_0 \cdot \mathscr{L}_\gamma(\omega) + (1 - A_0)\mathscr{L}_{\gamma+\Gamma}(\omega)\} \quad (1)$$

$$A_0 = p + (1-p) \cdot (\phi \cdot A_3 + (1-\phi) \cdot A_s) \quad (2)$$

$$A_3 = \frac{1 + 2 \cdot j_0(q \cdot a)}{3} \quad (3)$$

$$A_s = \left(\frac{3 \cdot j_1(qR)}{qR}\right)^2 \quad (4)$$

with $j_0(x)$ and $j_1(x)$ are the zeroth and first order spherical Bessel function of the first kind.[1,2] The q dependence of the EISF has been fixed using the parameters R=6.5 Å, p=0.28, $\phi = 0.55$, a=1.715 Å and is shown in Figure S1. The linewidths γ and Γ, describing the apparent

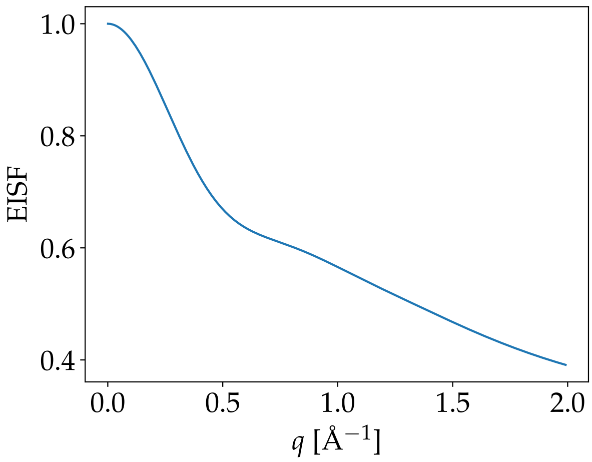

Figure S1: q dependence of the parametrized EISF based on Equation 2.

global and internal diffusion, were described with a fickian diffusion and jump diffusion process, respectively:

$$\gamma = Dq^2 \tag{5}$$

$$\Gamma = \frac{D_{int}q^2}{1 + D_{int}q^2\tau} \tag{6}$$

using $D = 2\,\text{Å}^2\mu\text{eV}$, $D_{int} = 35\,\text{Å}^2\mu\text{eV}$ and $\tau = 0.001\,\mu\text{eV}^{-1}$. The energy transfer $\hbar\omega$ dependence of the modeled spectra is shown for different momentum transfers q in Figure S2.

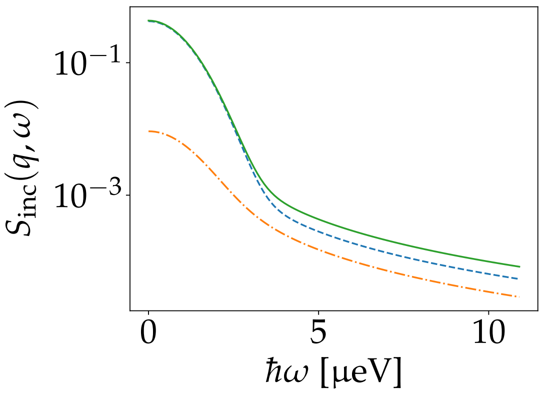

(a) $q = 0.1\text{Å}^{-1}$

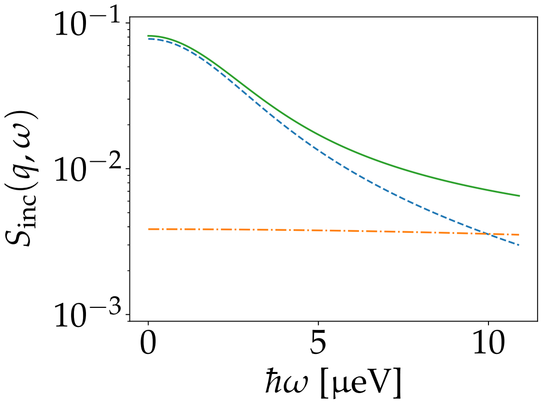

(b) $q = 1.0\text{Å}^{-1}$

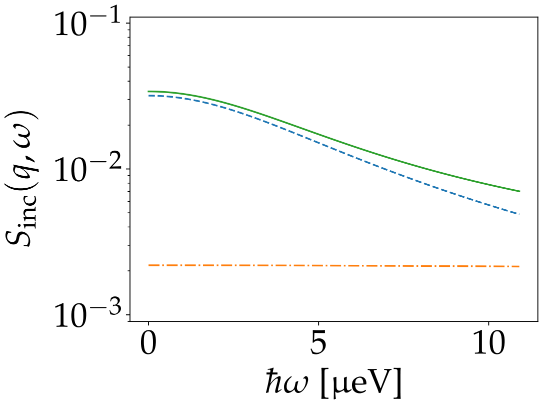

(c) $q = 1.5\text{Å}^{-1}$

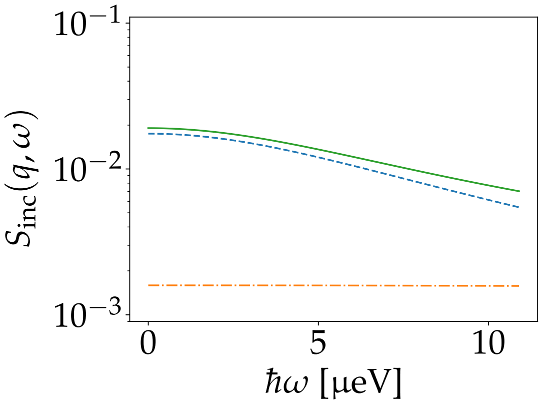

(d) $q = 1.9\text{Å}^{-1}$

Figure S2: Energy transfer $\hbar\omega$ dependence of the modeled spectra for different momentum transfers q. Blue dashed, orange dashed-dotted and green solid lines represent the contributions from the global and internal diffusion as well as the total incoherent scattering signal of the protein, respectively.

S2 Fit of experimental full QENS spectra

The contribution of the empty can as well as the solvent contribution have been subtracted from the full QENS spectra. These reduced QENS spectra were subsequently analyzed with a sum of two Voigt functions. The fits are shown for $q = 1\text{Å}^{-1}$ in Figure S3. The line widths γ are shown as a function of q^2 in Figure S4.

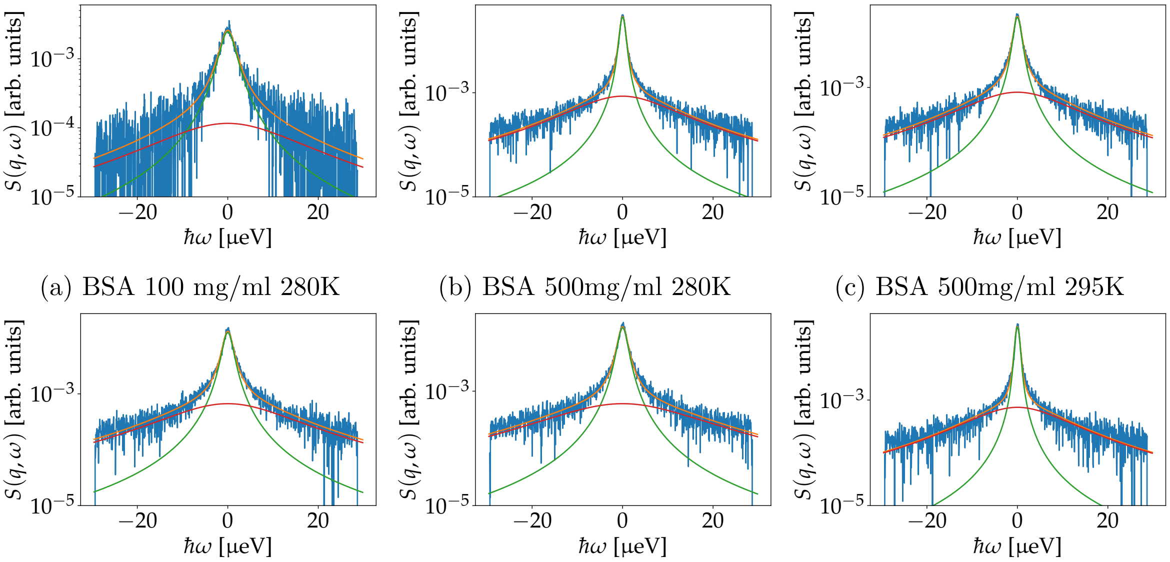

(a) BSA 100 mg/ml 280K

(b) BSA 500mg/ml 280K

(c) BSA 500mg/ml 295K

(d) BSA 500mg/ml 310K

(e) Myo 500 mg/ml 280K

(f) polyIg 500 mg/ml 280K

Figure S3: Example fits of the full QENS spectra using two Voigt functions. The solvent contribution and empty can contribution were subtracted prior to fitting. All plots are at $q = 1\text{Å}^{-1}$.

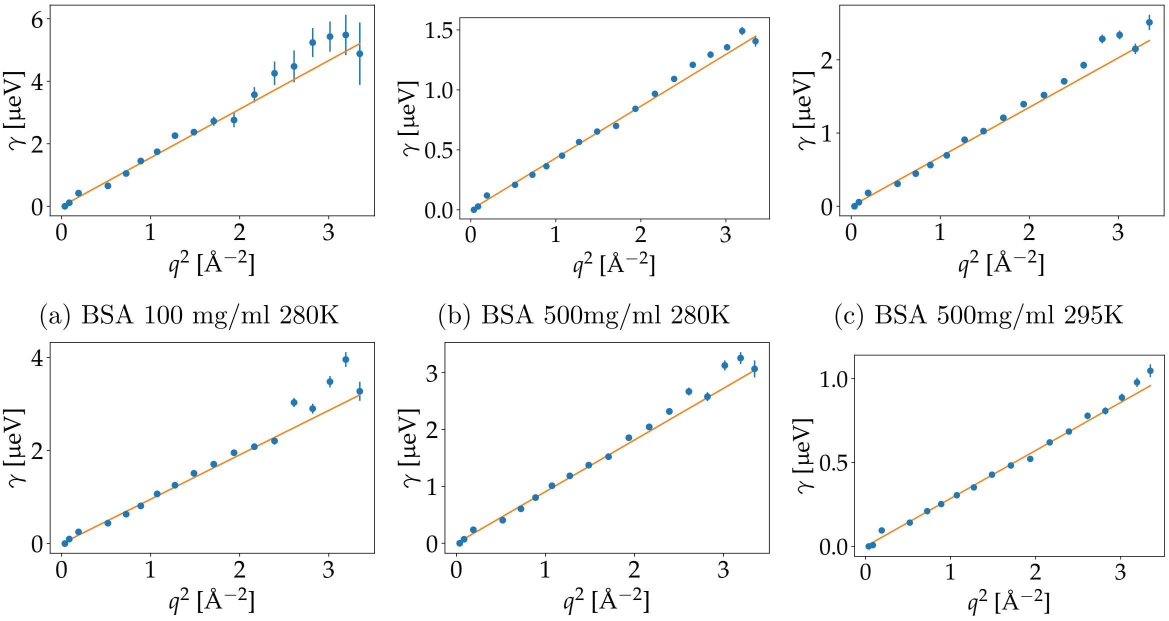

(a) BSA 100 mg/ml 280K
(b) BSA 500mg/ml 280K
(c) BSA 500mg/ml 295K
(d) BSA 500mg/ml 310K
(e) Myo 500 mg/ml 280K
(f) polyIg 500 mg/ml 280K

Figure S4: q- dependent line broadening of the thinner Voigt function attributed to the protein center-of-mass diffusion obtained from the full QENS fits for the protein solution samples investigated in this work.

S3 Energy-dependent flux impinging on the sample

Based on Figure 2b of the main text, the energy-dependent neutron flux from the primary spectrometer to the sample can be estimated, assuming the moving Doppler monochromator setup. In Figure S5, the flux distribution is shown as a function of energy transfer for the two operation modes, i.e., for the sinus velocity profile to record full spectra, and for the profile to record inelastic fixed-window scans where the monochromator displacement is linear in time outside the "turn-around" regions. Already in this simplified approach, it becomes visible that the FWS are characterized by a significantly higher flux for the given energy transfer.

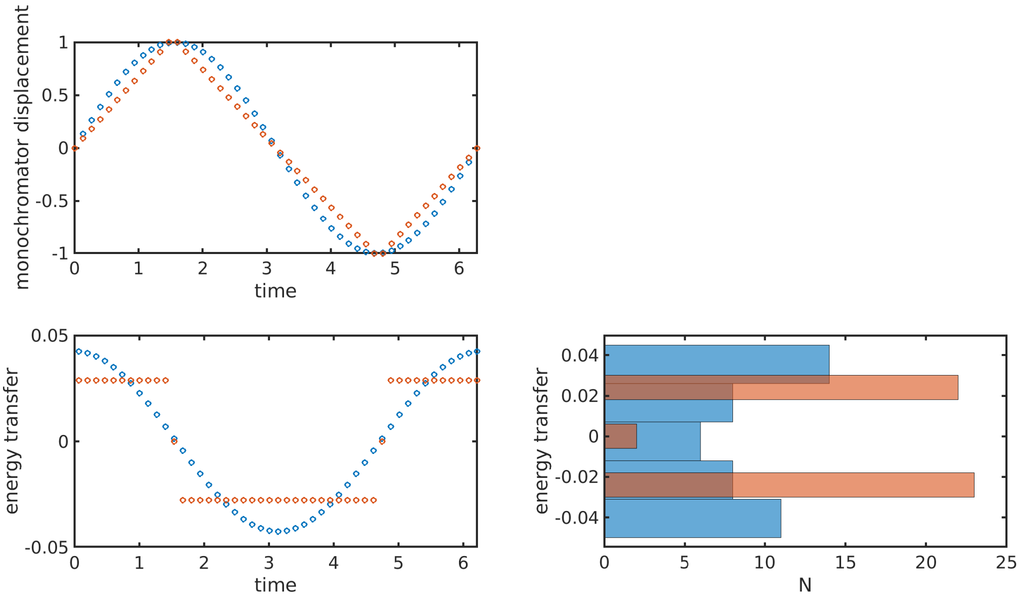

Figure S5: Derivation (left column) of the energy-dependent histogram of the neutron flux impinging on the sample (right column): For FWS (brown), the flux per energy transfer interval is significantly higher than for full QENS spectra (blue). All axes are in arbitrary units.

S4 Fit of sparse QENS spectra

The q dependence of the broadening of the second Lorentzian function of the sparse QENS is shown in Figure S6.

To investigate the robustness of the results, additional fits with only one single Lorentzian function have been carried out to model the $\hbar\omega$ dependence of the different samples (Figure S7). The fit results are summarized in Table S1.

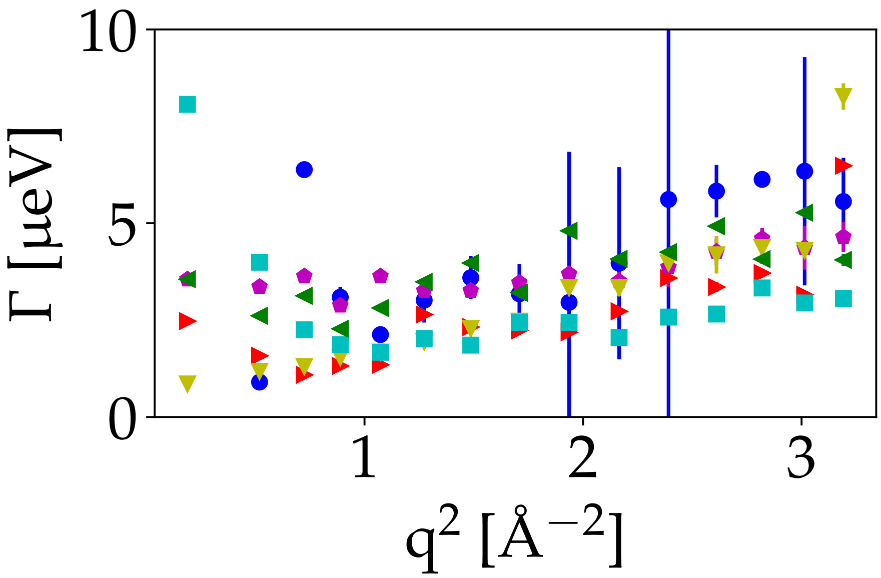

Figure S6: Broadening of the second Lorentizan function from the sparse QENS fits. Blue spheres, magenta pentagons, cyan squares, olive downwards pointing triangles, red right pointing triangles and green left pointing triangles represent values of BSA 100 mg/ml at 280 K, myoglobin 500 mg/ml at 280 K, polyclonal IgG 500 mg/ml at 280 K, BSA 500 mg/ml at 310 K, BSA 500 mg/ml at 295 K and BSA 500 mg/ml at 280 K, respectively.

Table S1: Effective diffusion coefficients D in Å^2/ns obtained from the broadening of the single Voigt function used to describe the data in Figure S7 for different maximum energy transfers $\hbar\omega_{max}$ of inelastic energy transfers for the different samples specified in the main text (Table 1).

	$\hbar\omega_{max}$ = 3 μeV	$\hbar\omega_{max}$ = 5 μeV	$\hbar\omega_{max}$ = 7 μeV	$\hbar\omega_{max}$ = 10 μeV
Sample 1	977.28±1260.41	2.80±0.29	2.91±0.28	2.94±0.23
Sample 2 280K	0.53±0.01	0.59±0.02	0.62±0.02	0.63±0.02
Sample 2 295K	1.01±0.04	1.13± 0.05	1.20±0.06	1.25±0.07
Sample 2 310K	1.43±0.09	1.65±0.07	1.78±0.08	1.90±0.10
Sample 3	1.15±0.06	1.43±0.08	1.55±0.10	1.65±0.12
Sample 4	0.36±0.02	0.39±0.02	0.41±0.02	0.41±0.02

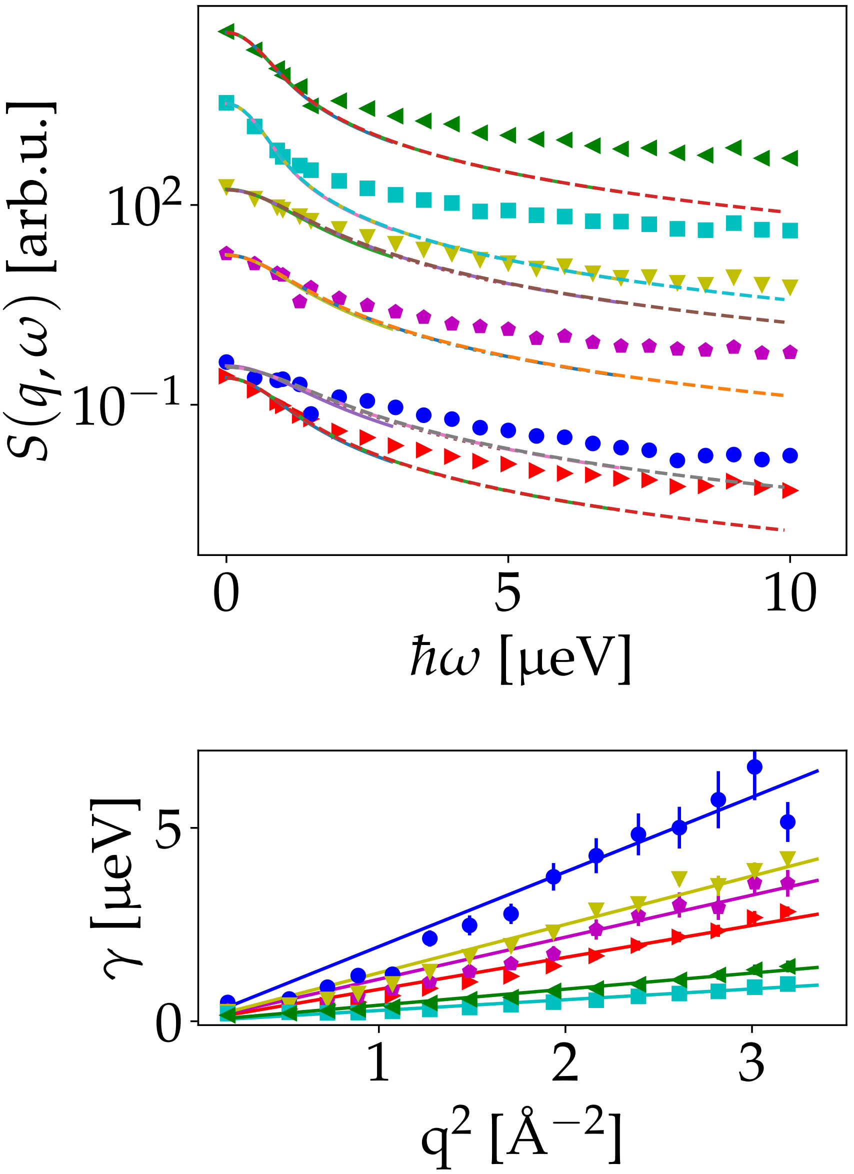

Figure S7: Sparse QENS analysis for different samples using one single Voigt function to fit the energy dependence. The top figure represents the fits at $q = 1$ $Å^{-1}$ for different maximum energy transfers $\hbar\omega_{max} = 3$ µeV (solid orange line), $\hbar\omega_{max} = 5$ µeV (green dashed line), $\hbar\omega_{max} = 7$ µeV (red dashed-dotted line), $\hbar\omega_{max} = 10$ µeV (violet dashed line). Different data sets are rescaled by a factor of 10 each for better visibility. The bottom figure shows the q^2 dependence of the corresponding widths as well as a fit of $\gamma = Dq^2$ to determine the diffusion coefficient D. In both plots, blue spheres, magenta pentagons, cyan squares, olive down pointing triangles, red right pointing triangles and green left pointing triangles represent values of BSA 100 mg/ml at 280 K, myoglobin 500mg/ml at 280 K, polyclonal IgG 500mg/ml at 280 K, BSA 500 mg/ml at 310 K, BSA 500 mg/ml at 295 K and BSA 500 mg/ml at 280 K, respectively.

S5 Dependence of $C(\gamma)$ on $^-h\omega_i$ and $^-h\omega_r$

In Figure S8, the calibration curve $C(\gamma)$ is shown for different energy transfers chosen for the FWS. It can be seen that the calibration curves cross at small γ. Since the resolution function is constant over time for an instrument, the analysis framework is not impacted by this effect as long as the calibration curve is calculated with the right energy transfers and the correct resolution. The best resolution is featured by a plateau. This observation highlights once more the importance of the right choice of the resolution function.

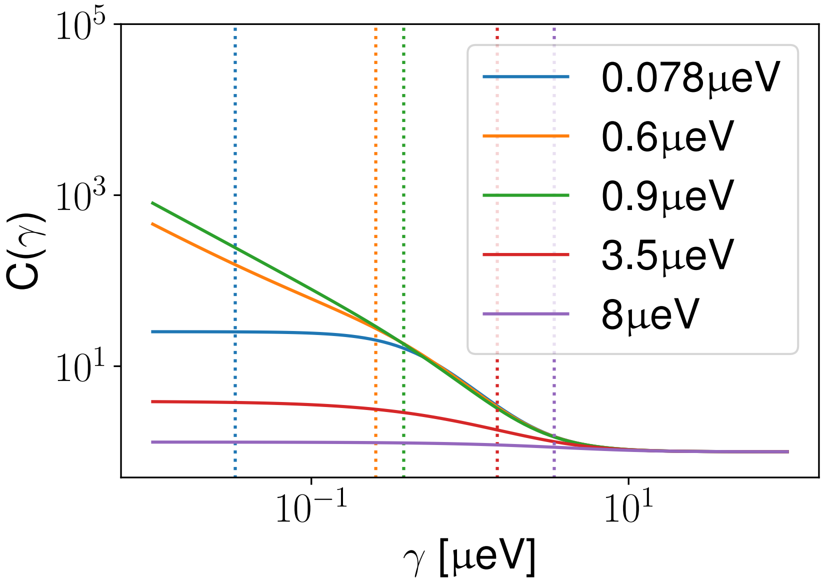

Figure S8: $C(\gamma)$ for different instrument resolutions with energy transfers $\hbar\omega_i = 0.5$μeV and $\hbar\omega_r = 2.5$μeV.

S6 Ratio Analysis Applying Two Gaussian Functions to Describe the Resolution Function

To investigate the influence of the description of the resolution function on the final fit quality, the resolution function has been approximated with two free gaussian functions to ensure a better description of the experimental data. Equation 8 from the main text then reads:

$$\begin{aligned} C(\gamma) &= \frac{\mathscr{R}(\hbar\omega_i) \otimes \mathscr{L}_\gamma(\hbar\omega_i)}{\mathscr{R}(\hbar\omega_r) \otimes \mathscr{L}_\gamma(\hbar\omega_r)} \\ &\overset{(1)}{=} \frac{a_1\mathscr{V}_{\sigma_1,\gamma}(\hbar\omega_i) + a_2\mathscr{V}_{\sigma_2,\gamma}(\hbar\omega_i)}{a_1\mathscr{V}_{\sigma_1,\gamma}(\hbar\omega_r) + a_2\mathscr{V}_{\sigma_2,\gamma}(\hbar\omega_r)} \end{aligned} \tag{7}$$

with $a_{1,2}$ and $\sigma_{1,2}$ being the scaling parameters and the widths of the two Gaussian functions, respectively. Figure S9 displays the R^2 values obtained from the analysis using two Gaussian functions for the resolution function as a function of the two different used energy transfers $\hbar\omega_{i,r}$. Only small improvements can be observed for the description using two Gaussian functions for the description of the resolution function.

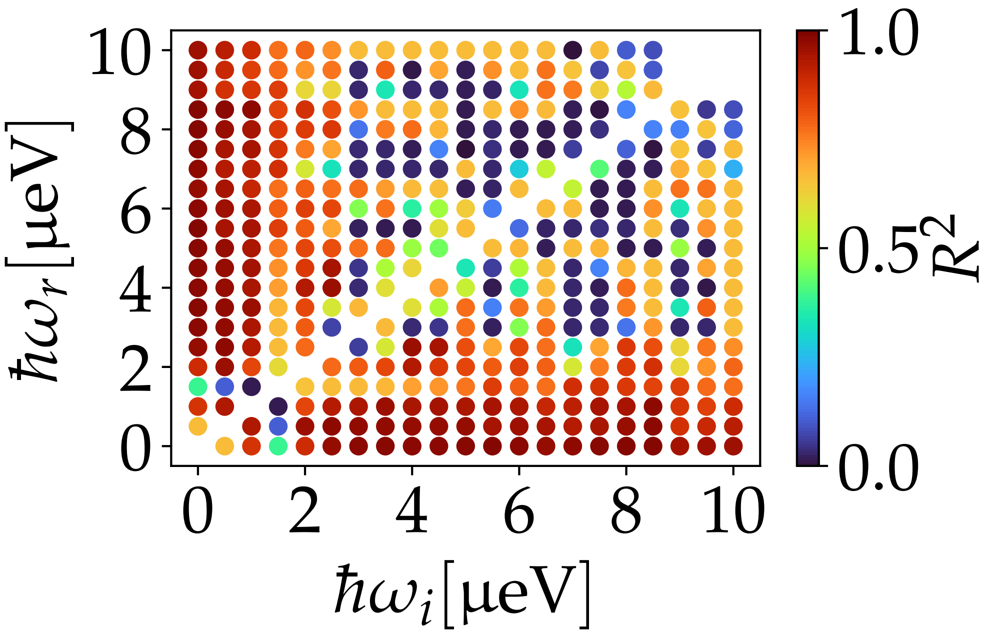

Figure S9: R^2 values comparing the fit results of the diffusion coefficients of different proteins obtained from full QENS analysis and the diffusion coefficients obtained from the ratio analysis using two Gaussian functions for the description of the resolution function.

S7 Alternative approach for the ratio analysis of powder samples

Since the QENS signal of powder samples is characterized by a significant elastic contribution and by an absence of center-of-mass diffusion, the approach applied in the main text might not be applicable. However, if the incoherent scattering is described by

$$S(q,\omega) = A_0\delta(\omega) + (1 - A_0)\mathscr{L}_\Gamma(\omega), \tag{8}$$

i.e., by equation (1) of the main text for $\gamma = 0$, the broadening Γ can be determined as described in the main text, based on two IFWS. The resulting q dependence of Γ is shown in Figure S10.

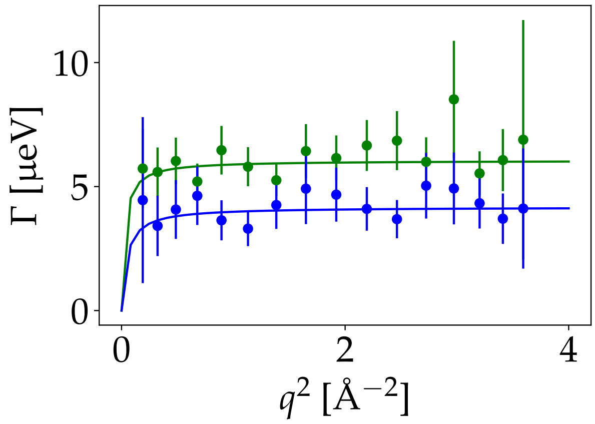

Figure S10: Γ as a function of q^2 of H_2O (green) and D_2O (blue) hydrated BSA powders characterizing the internal diffusive properties of the protein. IFWS with $\hbar\omega = 3$ µeV and $\hbar\omega = 6$ µeV have been used.

By taking the EFWS, the ratio r can be described by

$$r = \frac{S(q,\omega=0)}{S(q,\omega_i)} = \frac{A_0\mathscr{R}(0) + (1 - A_0)\mathscr{V}_\Gamma(0)}{(1 - A_0)\mathscr{V}_\Gamma(\omega)} \tag{9}$$

with $\omega_i \neq 0$. It is then possible to determine the elastic incoherent structure factor EISF $A_0(q)$:

$$A_0 = \frac{r\mathscr{V}_\Gamma(\omega) - \mathscr{V}_\Gamma(0)}{\mathscr{R}(0) - \mathscr{V}_\Gamma(0) + r\mathscr{V}_\Gamma(\omega)} \tag{10}$$

In Figure S11, the EISF determined with Equation 10 assuming an averaged q-independent Γ based on the values from Figure S10 is shown, by taking the average over the symbols in Figure S10. It should be emphasized that the EISF thus determined depends on the assumed value of Γ. Figure S12 depicts the EISF for different values of Γ. The value of Γ mainly affects the immobile fraction of the EISF, i.e. $A_0(q \to \infty)$. Figure S13 and Figure S14 show the EISF for different choices of Γ and the determined q dependence of Γ for the measurements performed on IN13, respectively.

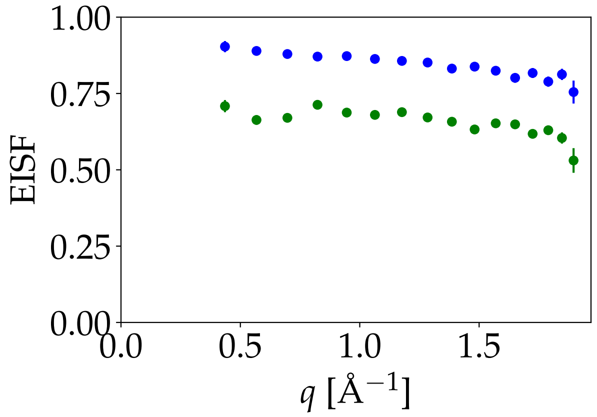

Figure S11: EISF determined by Equation 10. The EFWS and IFWS with $\hbar\omega$ = 6 μeV have been used in combination with the averaged value of Γ from Figure S10.

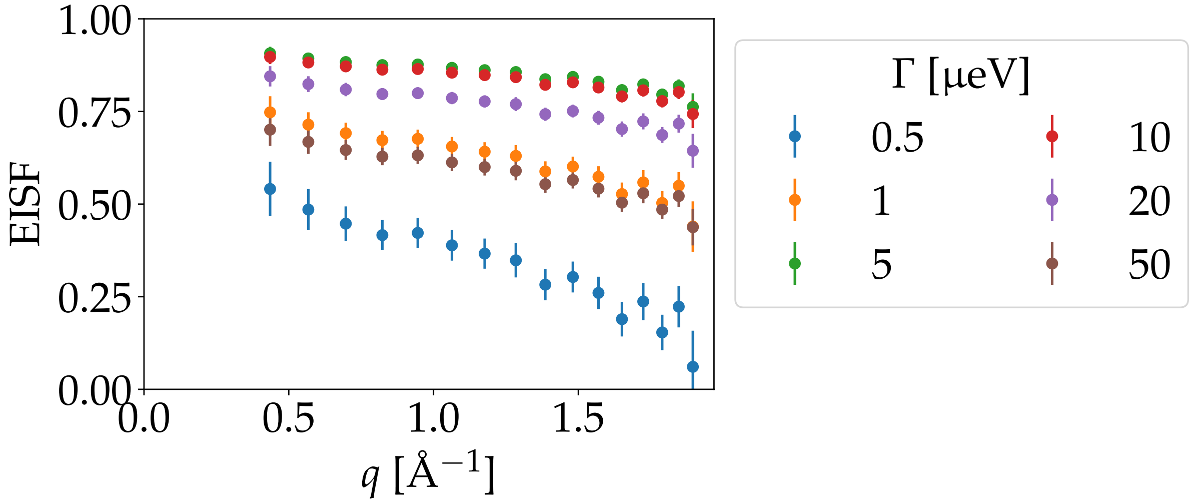

Figure S12: EISF for the D_2O hydrated BSA powder sample determined based on Equation 10 assuming different values of Γ as indicated in the legend.

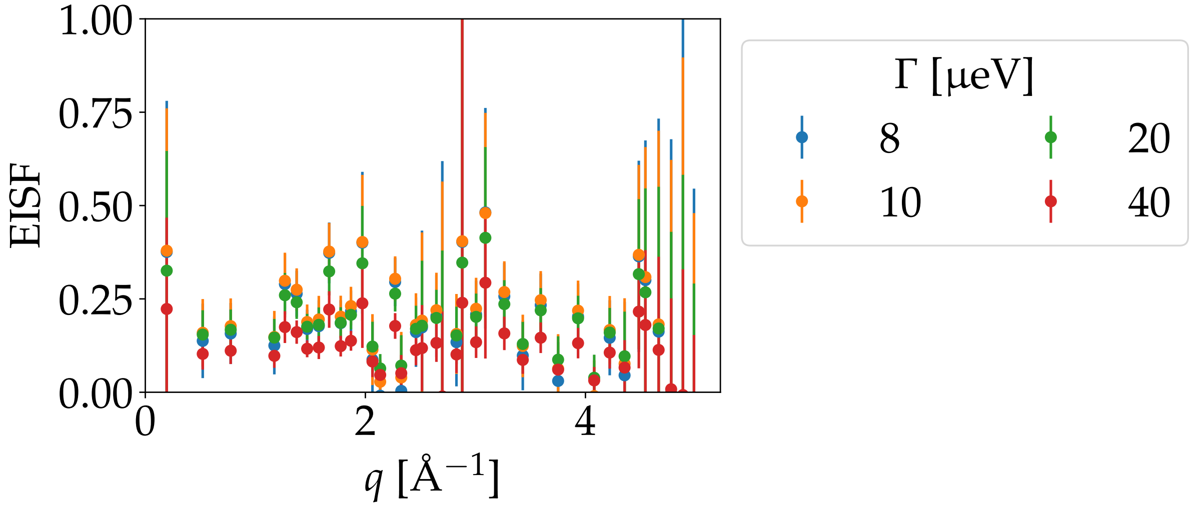

Figure S13: EISF determined from the H_2O hydrated BSA powder for different choices of Γ. Although the 3μeV offset is still within the resolution function of the instrument, an extraction of the EISF is not possible anymore.

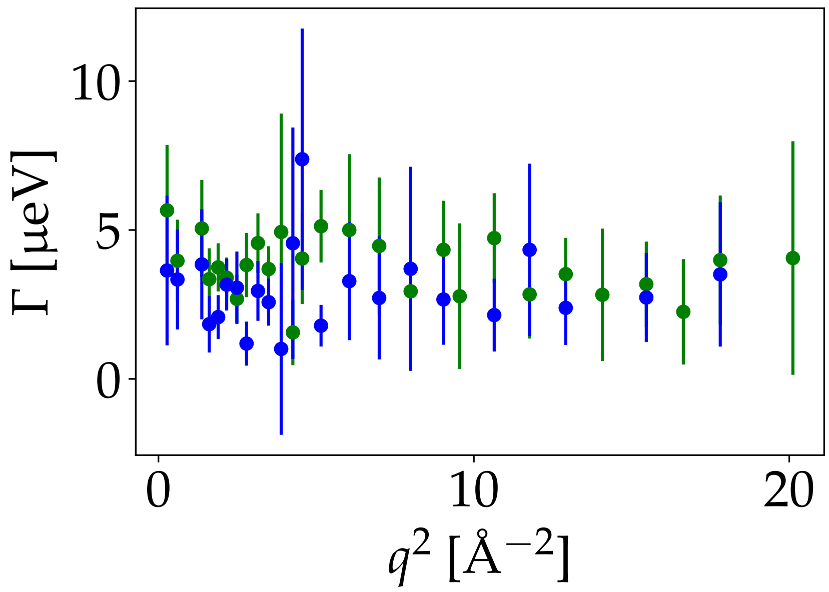

Figure S14: Γ as a function of q of H_2O (green) and D_2O (blue) hydrated BSA powders determined by the ratio analysis using the FWS recorded at $\hbar\omega_i = 3$ μeV and $\hbar\omega_r = 9$ μeV on IN13.
